# Social status predicts physiological and behavioral responses to chronic stress in rhesus monkeys

**DOI:** 10.1016/j.isci.2024.110073

**Published:** 2024-05-21

**Authors:** Zhiyi Zhang, Xueda Dong, Zhiqiang Liu, Ning Liu

**Affiliations:** 1 State Key Laboratory of Brain and Cognitive Science, Institute of Biophysics, Chinese Academy of Sciences, Beijing 100101, China; 2 Sino-Danish College, University of Chinese Academy of Sciences, Beijing 100049, China; 3 College of Life Sciences, University of Chinese Academy of Sciences, Beijing 100049, China

**Keywords:** ecology, behavioral neuroscience, social sciences, psychology

## Abstract

Investigating the underlying factors that cause differential individual responses to chronic stress is crucial for developing personalized therapies, especially in the face of pandemics such as COVID-19. However, this question remains elusive, particularly in primates. In the present study, we aimed to address this question by utilizing monkeys as a model to examine the impacts of social rank on stress levels and physiological and behavioral responses to chronic stress primarily caused by social isolation at both the individual and group levels. Our results showed that high-ranking animals were more susceptible to chronic stress. After exposure to chronic stress, although social hierarchies remained the same, the colonies exhibited more harmonious group relationships (e.g., more prosocial behaviors), with notable contributions from low-ranking animals. Overall, this study deepens our understanding of how social status shapes responses to chronic stress and sheds light on developing tailored and personalized therapies for coping with chronic stress.

## Highlights

• High-ranking animals were more susceptible to chronic stress
• Social rank influenced physiological/behavioral responses to chronic stress
• The colonies exhibited more harmonious group relationships after chronic stress
• Oxytocin and social support may play a critical role in coping with chronic stress

## Graphical abstract

**Figure:**
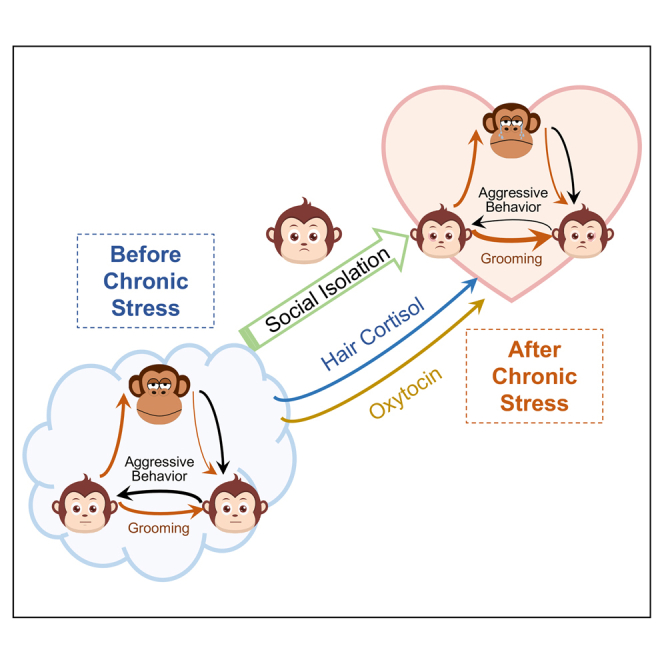

## Introduction

A growing body of evidence has demonstrated that chronic stress plays a critical role in the onset and development of various disorders (for a review, see Kivimäki et al.^1^). Various pressures in life can serve as sources of chronic stress. In social species, such as humans, social interactions are essential to the health and survival of social animals, similar to necessities such as food and water.^2^ As a result, social isolation or loneliness is a significant source of chronic stress,^3^^,^^4^ such as that experienced during the COVID-19 pandemic with the necessity for social distancing and reduction in social interactions (e.g., quarantine).^5^^,^^6^ When the intensity and duration of stress surpass an individual’s capacity to cope, detrimental effects may be induced on both physical and mental well-being. Individuals vary in their responses to chronic stress, with some rapidly recovering while others do not.^7^ Revealing the factors that underlie differential individual responses to chronic stress will aid in developing tailored and personalized therapies for stress-related disorders.

For social animals, social status is essential to individual differences^8^^,^^9^ and may shape how individuals respond to environmental challenges, including chronic stress. Previous studies have established a connection between social status and health outcomes in humans and other social species.^10^^,^^11^^,^^12^ An individual’s social status can predict disease risk and mortality.^13^ For example, higher socioeconomic status is associated with increased longevity, heightened well-being, and reduced incidence of stress-related diseases.^12^^,^^14^ These health benefits may be partially attributable to lower stress levels associated with higher social status.^15^^,^^16^ The aforementioned evidence raises a critical question regarding whether social status may also influence how people experience and cope with chronic stress. However, few studies (especially in humans) have explored the potential link between social status and responses to chronic stress.^17^^,^^18^^,^^19^

Despite limited animal model studies suggesting that social status may influence an individual’s perception and response to chronic stress,^20^^,^^21^ conclusions remain inconsistent. For example, some studies indicated dominant mice’s susceptibility to chronic social defeat stress,^22^ whereas other studies suggested that subordinates were more susceptible,^23^^,^^24^ and some studies reported no correlation between social hierarchy and chronic social defeat stress.^25^ It is worth noting that these studies have been primarily conducted in rodents, whose social behaviors are less complex than those in humans. Non-human primates exhibit similar complex social and stress-related behaviors to humans and display social status that more closely resembles that of humans in well-established social hierarchies.^26^ Therefore, non-human primates provide an ideal model for exploring the relationship between an individual’s social rank and responses to chronic stress.

Previous studies have investigated responses to chronic stress in various non-human primates. As in humans, chronic mild stress leads to depressive- and anxiety-like behaviors, as well as disease risk in monkeys.^27^^,^^28^^,^^29^ Nevertheless, the potential influence of social rank on responses to chronic stress remains poorly explored. Limited evidence implies the possible relationship between social status and responses to chronic stress. For example, one study reported that dominant monkeys exhibit higher heart rates than subordinate monkeys during chronic stress.^27^ In addition, studies in non-human primates (and rodents) have primarily focused on individual responses to chronic stress, neglecting adaptation to chronic stress at the group level, for example, the impact of chronic stress on social structure and relationships. Understanding group-level responses to chronic stress may provide new insights into the prevention and treatment of stress-related disorders, especially when a group is exposed to chronic stress at the same time, such as that experienced during the COVID-19 pandemic.

In the present study, we used monkeys as a model to examine the impact of social rank on stress levels induced primarily by social isolation as well as physiological (e.g., immune and hormonal) and behavioral responses to stress at both the individual and group levels. We hypothesized that an individual’s social status predicts physiological and behavioral responses to chronic stress.

## Results

**Figure 1:**
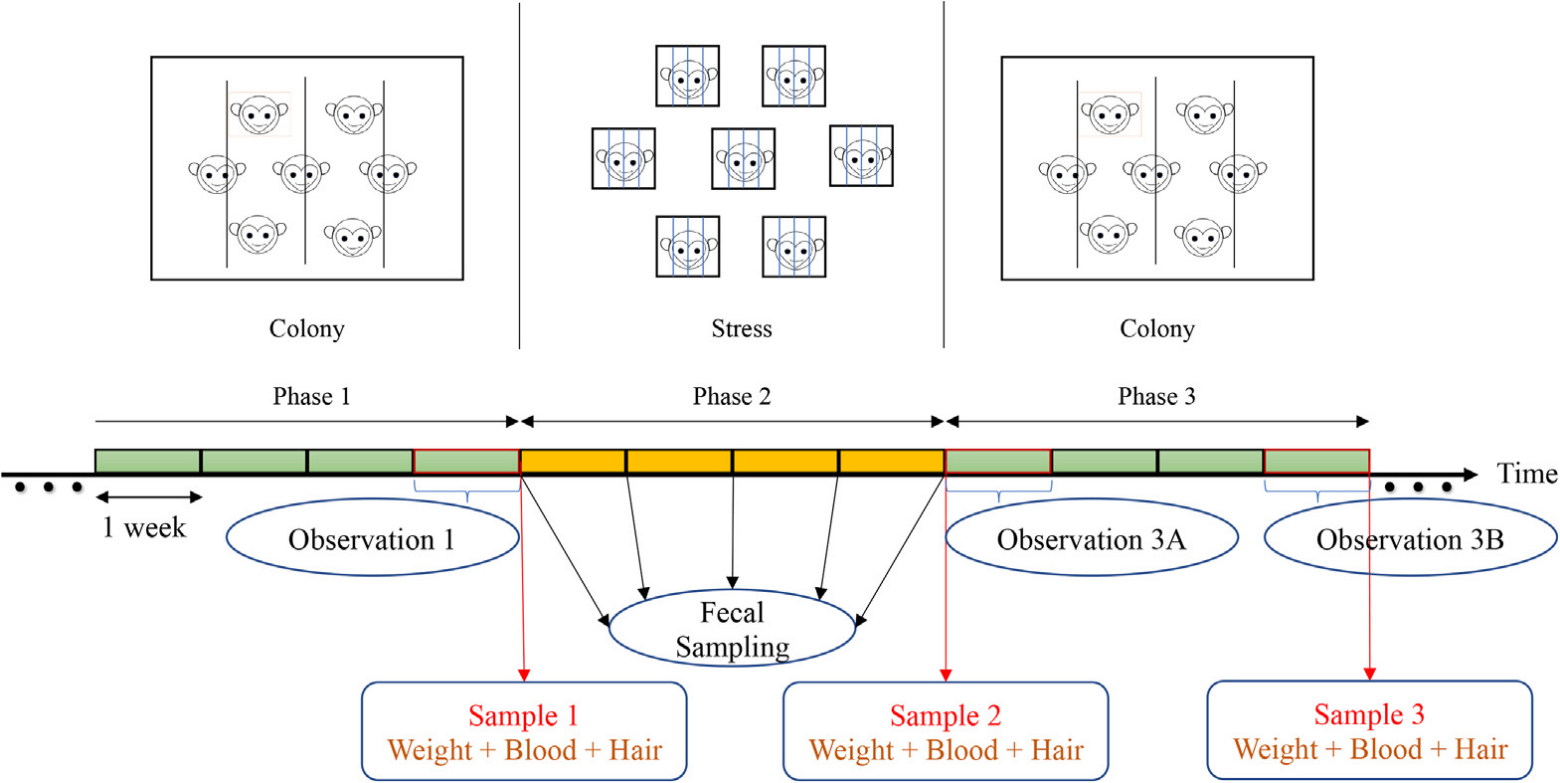
Experimental procedures In Phase 1, monkeys were housed in their original home cages. At the onset of Phase 2, all animals were transferred into single cages within the same room. Subjects remained in olfactory and auditory but not visual or tactile contact with others. Moreover, one of two unpredictable stressors was applied once per day: space restriction and intimidation. Four weeks later, all the monkeys were returned to their original home cages (Phase 3). Weight, blood and hair samples were collected at the end of Phase 1, Phase 2, and Phase 3, corresponding to Sample 1, 2, and 3, respectively. Fecal samples were collected once animals were transferred into single cages and then at one-week intervals in Phase 2, resulting in a total of five samples per monkey. Behavioral data were collected at the end of Phase 1, in the first week (Observation 3A) and last week (Observation 3B) of Phase 3.

The present study was conducted on two groups of adult female macaques, designated as Group B](Beijing, n = 7) and Group SZ (Suizhou, n = 6), and comprised three phases (Figure 1). In Phase 1, the monkeys were housed in their original home cages, then transferred to single cages within the same room and raised there for four weeks (Phase 2), and finally returned to their original home cages (Phase 3).

### Effects of chronic stress on social hierarchy

Based on David’s score (DS),^30^ we calculated the social rank of each monkey in each group at three observation periods (i.e., Observation 1, 3A, and 3B). To account for the discrepancy in the number of animals between the two groups, we normalized DS using Equations 1-1 and 1-2, as described in previous studies.^31^

(Equation 1-1) $${DS}_{i}^{\prime }={DS}_{i}+|{DS}_{\min}|$$

(Equation 1-2) $${NormDS}_{i}=\frac{{DS}_{i}^{\prime }}{{DS}_{\max}^{\prime }}$$

where ${DS}_{i}$ represents the original DS for a specific monkey i, ${DS}_{\min}$ is the minimum value of DS within the given group, and ${DS}_{\max}^{\prime }$ is the maximum value of DS within the same group after applying the transformation described by Equation 1-1. Through this normalization process, we assigned the highest-ranking individual within each group a score of 1, while the lowest-ranking individual received a score of 0.

**Figure 2:**
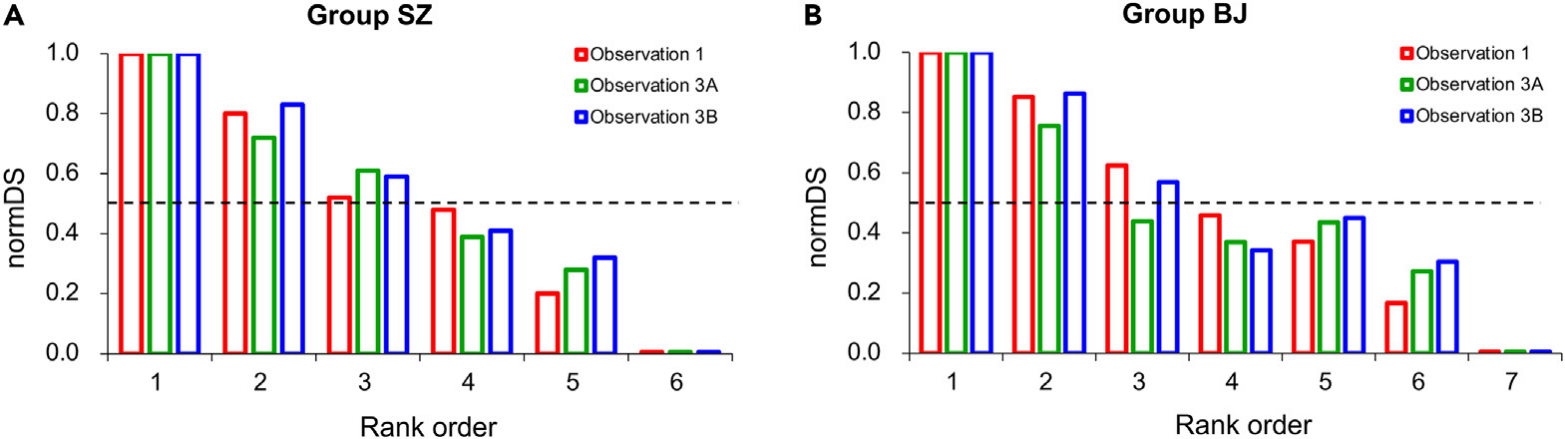
Social hierarchies before (Observation 1) and after chronic stress (Observation 3A and 3B) (A) and (B) show data from Group SZ and Group BJ, respectively. Dashed lines indicate normalized David’s score = 0.5. Note that the lowest-ranked animals (specifically Rank 6 in the SZ group and Rank 7 in the BJ group) had a standardized David score (normDS) of zero.

Our results showed no obvious changes in social hierarchy after social isolation (Figure 2), although the social ranks of two low-ranking (LR) individuals (#4 and #5) switched in Group BJ (Figure 2B). To facilitate comparisons, we used the initial (Observation 1) social ranks in the following analyses.

To assess potential differences in stress levels as well as physiological and behavioral responses between high-ranking (HR) and LR animals, we divided each social group into HR (DS > 0.5) and LR (DS < 0.5) based on social rank. Then, we conducted the two-way repeated measures analysis of variance (ANOVA) with time (n = 3 for weights, blood and hair samples, and behavioral data; n = 5 for fecal samples; Figure 1) as the within-subjects factor and rank (HR and LR) as the between-subjects factor.

### Stress levels after chronic stress

**Figure 3:**
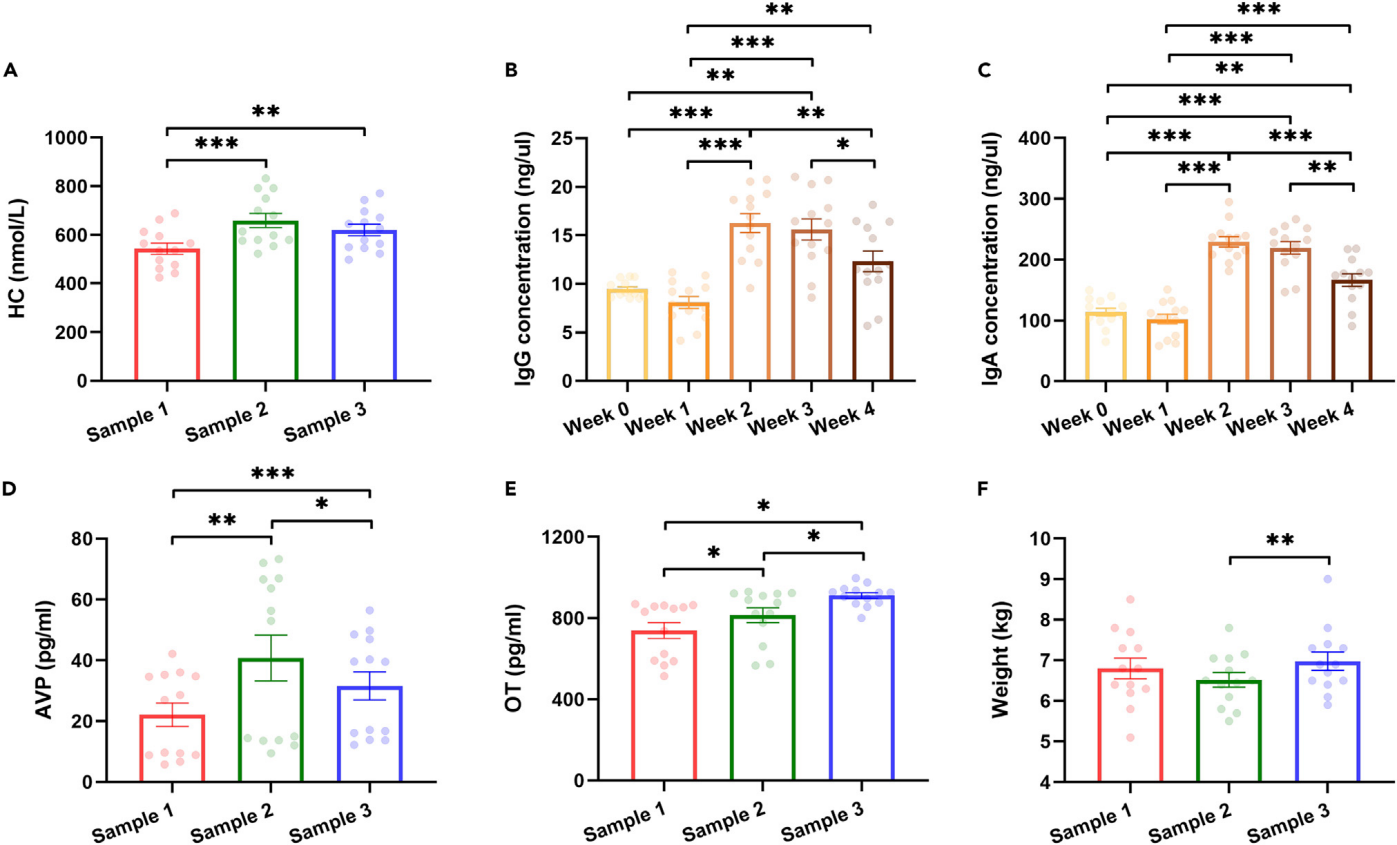
Physiological levels before, during, and after chronic stress (A) shows HC levels before and after chronic stress. (B and C) show IgG and IgA levels during chronic stress. (D–F) show AVP, OT, and weight levels before and after chronic stress, respectively. Black ∗*p* < 0.05, ∗∗*p* < 0.01, ∗∗∗*p* < 0.001 corrected. Error bars indicate standard error. Individual data points are shown in corresponding colors.

To minimize the risk of potential suffering, we implemented a shorter chronic stress period compared to previous studies.^27^^,^^29^^,^^32^ At first, to evaluate stress levels after chronic stress, we investigated changes in levels of hair cortisol (HC), a reliable biomarker of chronic stress in primates and other species.^33^^,^^34^ Our results showed a significant main effect of time on HC levels (Table 1). *Post hoc* tests showed that HC levels significantly increased after chronic stress (Sample 2vs1: *p* < 0.001), with no significant recovery after four weeks of social housing (Sample 3vs1: *p* = 0.006; Sample 3vs2: *p* = 0.089) (Figure 3A). These findings suggested that chronic stress was successfully induced in the present study and that the impacts of chronic stress persisted even after the monkeys were returned to social housing for four weeks. Next, we investigated the impact of chronic stress on physiological responses.

**Table 1:** Results of two-way repeated measures ANOVAs with Time as the within-subjects factor and Rank as the between-subjects factor for stress levels as well as immune and hormonal responses to chronic stress

Measurement	Main Effect of Time			Main Effect of Rank			Interaction		
	F	*p*	Partial η2	F	*p*	Partial η2	F	*p*	Partial η2
HC	15.408	<0.001^∗∗∗^	0.583	0.272	0.613	0.024	5.277	0.013^∗^	0.324
IgG	30.851	<0.001^∗∗∗^	0.737	0.141	0.714	0.013	0.677	0.611	0.058
IgA	72.393	<0.001^∗∗∗^	0.868	0.093	0.766	0.008	0.457	0.766	0.040
AVP	15.550	0.001^∗∗^	0.586	0.043	0.839	0.004	0.882	0.382	0.074
OT	9.895	<0.001^∗∗∗^	0.474	0.040	0.845	0.004	0.553	0.583	0.048
Weight	4.805	0.019^∗^	0.304	1.518	0.244	0.121	0.177	0.839	0.016

∗*p* < 0.05, ∗∗ *p* < 0.01, ∗∗∗*p* < 0.001.

### Physiological responses during and after chronic stress

Next, we investigated physiological (i.e., immune and hormonal) responses to chronic stress. To avoid introducing stressors caused by sample collection, only fecal samples were collected at one-week intervals during social isolation. As stress may lead to an increase in immune response,^35^^,^^36^ we tracked changes in immune response based on fecal levels of immunoglobulin G (IgG) and immunoglobulin A (IgA) during the period of social isolation (Phase 2) to monitor the impact of chronic stress on immune responses (Figures 3B and 3C). We found significant main effects of time on both IgG and IgA (Table 1). *Post hoc* tests revealed that levels of IgG and IgA were significantly elevated since Week 2 (IgG: Week 1vs0, *p* = 0.143; Week 2vs0, *p* < 0.001; Week 3vs0, *p* = 0.001; IgA: Week 1vs0, *p* = 0.234; Week 2vs0, *p* < 0.001; Week 3vs0, *p* < 0.001) and then started to recover at the end of social isolation (Week 4) (IgG: Week 4vs3, *p* = 0.026; IgA: Week 4vs3, *p* = 0.006), although not fully (IgG: Week 4vs0, *p* = 0.089; IgA: Week 4vs0, *p* = 0.003). These findings suggested that immune responses were initiated after two weeks of social isolation, followed by gradual adaptation.

Previous studies have reported that plasma oxytocin (OT) and arginine vasopressin (AVP) may be involved in stress response/regulation.^37^^,^^38^ Therefore, we also analyzed changes in plasma OT and AVP levels after chronic stress. We found significant main effects of time on these two hormones (Table 1). As shown in Figures 3D and 3E, OT and AVP significantly increased after chronic stress (Phase 2vs1: OT, *p* = 0.045; AVP, *p* = 0.002). After four weeks of social housing, levels of AVP recovered (Phase 3vs2: *p* = 0.030), albeit not fully (Phase 3vs1: *p* < 0.001), whereas OT continued to increase (Phase 3vs2: *p* = 0.039; Phase 3vs1: *p* = 0.010).

We also monitored animals’ weights throughout the experiment and found a significant main effect of time (Table 1): animals’ weights did not significantly decrease after chronic stress (Sample 2vs1: *p* = 0.268) but increased after four weeks of social housing (Sample 3vs2: *p* = 0.003; Sample 3vs1: *p* = 0.293) (Figure 3F). Since there were no significant changes after chronic stress, we did not perform further analysis on the animals’ weights.

### Relationships between stress levels and social hierarchy

**Figure 4:**
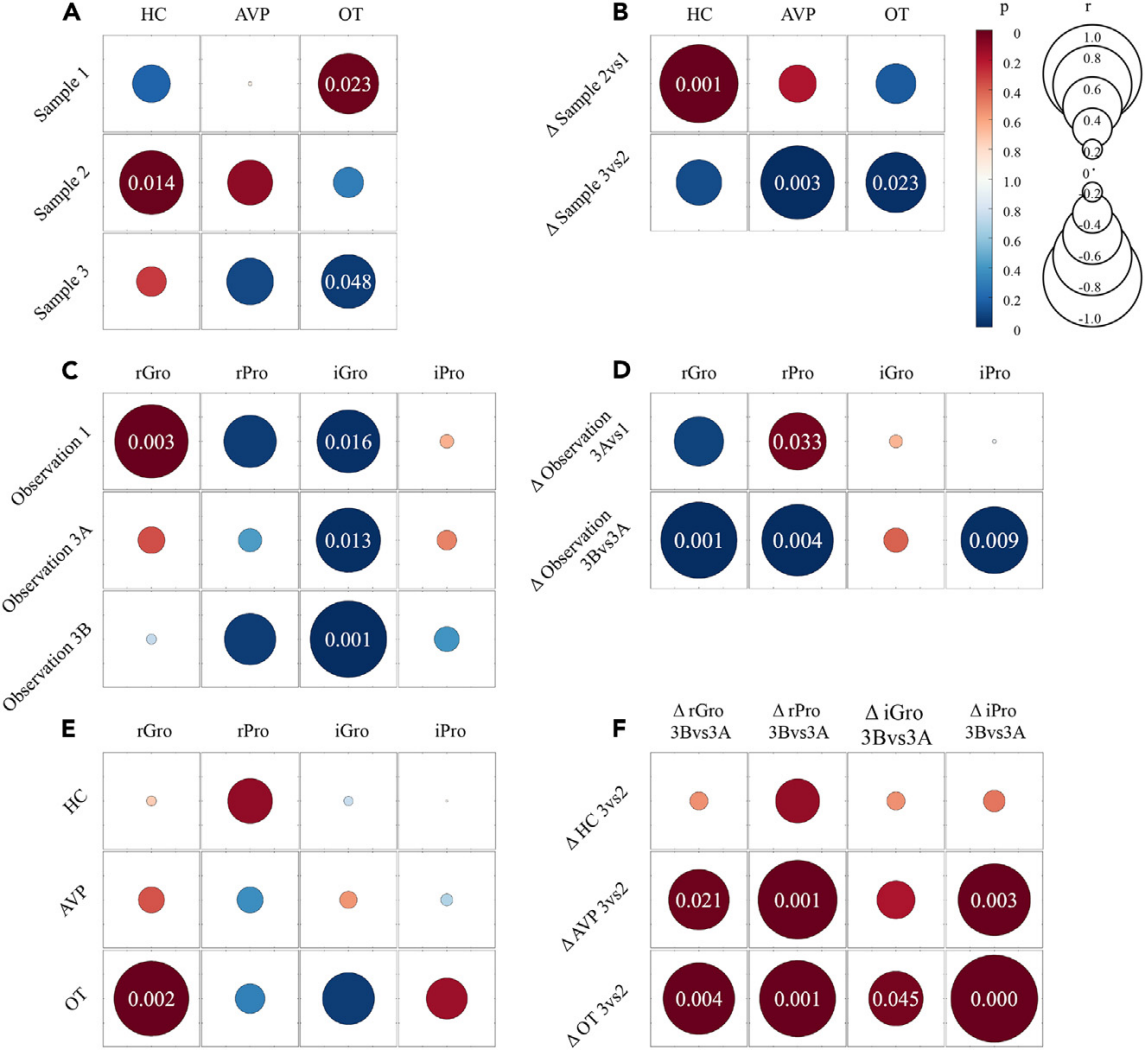
Relationships among social rank, hormones, and prosocial behaviors (A) shows relationships between social rank and hormones. (B) shows relationships between social rank and changes in hormones after chronic stress. (C) shows relationships between social rank and prosocial behaviors. (D) shows relationships between social rank and changes in prosocial behaviors after chronic stress. (E) shows relationships between hormones and prosocial behaviors in Phase 1. (F) shows relationships between changes in hormones and changes in prosocial behaviors during four weeks of reintroduction to social housing. A red circle indicates a positive correlation coefficient and a blue circle indicates a negative one. The color intensity of the circle indicates the *p* value (two-tailed). If the *p* value is less than 0.05, the *p* value is added to the circle. A full circle corresponds to r = 1 or −1 and an empty circle corresponds to r = 0. Gro: grooming, Pro: proximity.

**Figure 5:**
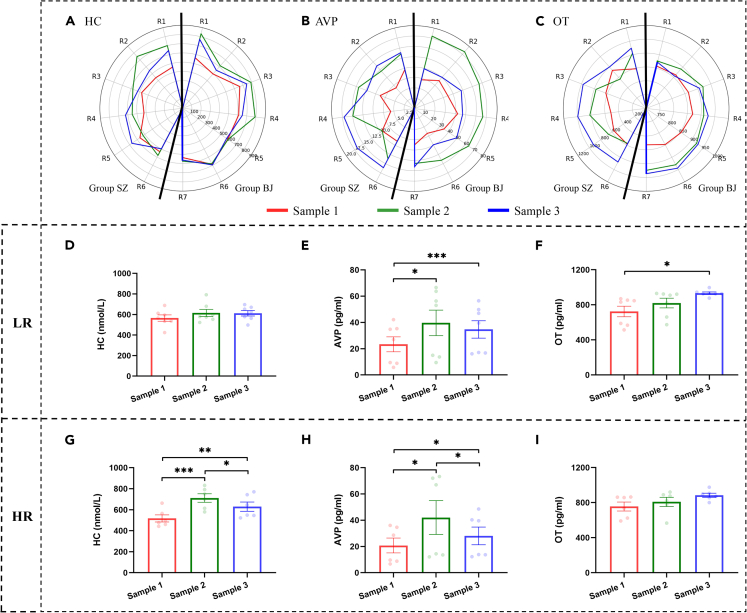
Hormone levels before and after chronic stress in high-ranking (HR) and low-ranking (LR) animals (A–C) show hormone levels at the individual level before and after chronic stress. (D–F) show HC, AVP, and OT levels in LR animals before and after chronic stress, respectively. (G–I) show HC, AVP, and OT levels in HR animals before and after chronic stress, respectively. Black ∗*p* < 0.05, ∗∗*p* < 0.01, ∗∗∗*p* < 0.001 corrected. Error bars indicate standard error. Individual data points are shown in corresponding colors. R1-R7 represent individuals from high to low social rank.

No significant correlation was found between social rank and HC levels during normal social life (Sample 1) (Figure 4A, HC – Sample 1), suggesting that stress levels were comparable across individuals in the observed groups before chronic stress. After social isolation (Sample 2), however, we detected higher HC levels in higher-ranking individuals (Figure 5A). Further analyses confirmed the significant positive correlation between social hierarchy and HC levels (Figure 4A, HC – Sample 2). Additionally, the increase in HC levels was also significantly positively correlated with social hierarchy (Figure 4B, HC – Δ Sample 2vs1), indicating that HR animals were more affected by chronic stress than LR ones. The two-way ANOVA also revealed a significant interaction between time and rank (Table 1). Our results showed that HC was significantly increased in HR animals (*p* < 0.001) but not in LR animals (*p* = 0.388) after chronic stress (Sample 2 vs. Sample 1) (Figures 5D and 5G). Furthermore, HC was significantly decreased in HR animals (*p* = 0.031) but not in LR animals (*p* = 0.938) after four weeks of reintroduction to social housing (Sample 3 vs. Sample 2). Note that, after four weeks of social housing, the relationship between HC levels and social rank showed a recovery: not as significant as before chronic stress (Figure 4A, HC – Sample 3).

### Relationships between physiological responses to chronic stress and social hierarchy

To explore the possible influence of individual rank in a social group on immune responses to stress caused by social isolation, we first analyzed the associations between social hierarchy and levels of IgG/IgA before isolation (Figure S1, Week 0 row). There were no significant correlations between social hierarchy and baseline (Week 0) levels of these immunoglobulins (IgG: r = 0.106, *p* = 0.730; IgA: r = 0.276, *p* = 0.361), indicating that individuals with different social ranks demonstrated comparable immune responses during normal social housing. We then investigated the relationship between social hierarchy and increases in IgG and IgA, especially peak changes (i.e., Δ Week 2vs0 and Δ Week 3vs0) (Figure S1, Δ Week 2vs0 and Δ Week 3vs0 rows). A significant negative correlation was detected for IgG (Δ Week 3vs0: r = −0.618, *p* = 0.024) but not for IgA, indicating that, during chronic stress, the higher the social rank, the less IgG increased and the fewer immune responses were induced.

Next, we investigated relationships between AVP and social hierarchy before and after chronic stress. During normal social housing, AVP levels were not significantly associated with social rank (Figure 4A, AVP – Sample 1) as HC levels. After chronic stress, AVP levels and their changes did not significantly correlate with social rank (Figure 4A, AVP – Sample 2; Figure 4B, AVP – Δ Sample 2vs1). However, a significant correlation between decreases in AVP after four weeks of social housing and social rank was observed (Figure 4B, AVP – Δ Sample 3vs2): the higher the social rank, the greater the decrease in AVP levels. Although the interaction between time and rank in the two-way ANOVA was not significant (Table 1), we presented *post hoc* results to provide a complete picture of AVP changes along with changes in HC: as HC levels, the decrease in AVP levels after four weeks of reintroduction to social housing was significant in HR animals but not in LR ones (Figures 5E and 5H).

We also investigated the relationships of OT levels with social hierarchy and found opposite patterns compared to HC. Before chronic stress, OT levels were significantly correlated with social rank (Figure 4A, OT – Sample 1), suggesting that, the higher the social rank, the higher the levels of OT. After chronic stress, the relationship between OT levels and social rank vanished (Figure 4A, OT –Sample 2). The changes in OT levels after chronic stress did not show a significant negative correlation with social rank (Figure 4B, OT – Δ Sample 2vs1). Notably, OT levels continued to increase during four weeks of social housing (Figure 3E). Therefore, we further investigated changes in OT after social housing (Δ Sample 3vs1) and did find that the aforementioned negative correlation with social rank became significant (*p* = 0.021). Moreover, the increase in OT levels during four weeks of reintroduction to social housing was negatively correlated with social rank (Figure 4B, OT – Δ Sample 3vs2): the higher the rank, the less the increase in OT levels. Consequently, the relationship between OT levels and social rank reversed from a positive correlation before chronic stress to a negative correlation after four weeks of reintroduction to social housing (Figure 4A, OT – Sample 3). The two-way ANOVA analysis in OT did not reveal a significant interaction between time and rank (Table 1). Again, to provide a complete picture of OT changes along with changes in HC, we conducted *post hoc* tests and found marginally significant changes in LR (*p* = 0.070) but not in HR animals during four weeks of social housing (Figures 5F and 5I).

### Changes in social behaviors and relationships after chronic stress

**Figure 6:**
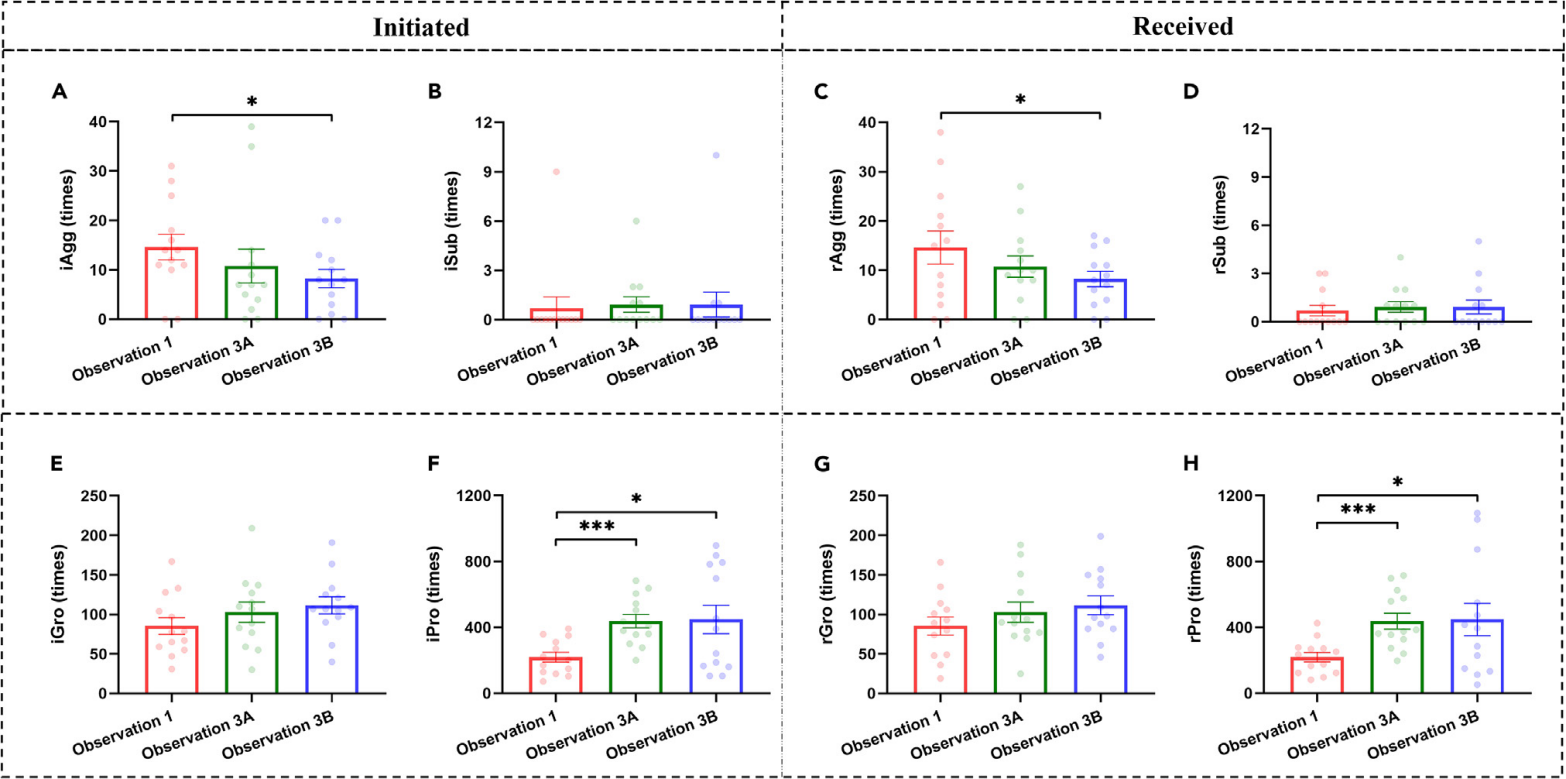
Social behaviors before and after chronic stress (A and B) show data of initiated aggressive and submissive behaviors, respectively. (C and D) show data of received aggressive and submissive behaviors, respectively. (E and F) show data of initiated grooming and proximity, respectively. (G and H) show data of received grooming and proximity, respectively. Black ∗*p* < 0.05, ∗∗∗*p* < 0.001 corrected. Error bars indicate standard error. Individual data points are shown in corresponding colors. Agg: aggressive behaviors, Sub: submissive behaviors, Gro: grooming, Pro: proximity.

To clarify the impact of chronic stress on social behaviors, we explored changes in frequencies of different types of social behaviors (i.e., aggressive behaviors [Agg], submissive behaviors [Sub], grooming [Gro], and proximity [Pro]). For each type of behavior, initiated (i.e., iAgg, iSub, iGro, and iPro) and received ones (i.e., rAgg, rSub, rGro, and rPro) were the same in the overall amount and showed similar results (Figure 6; Table 2). Therefore, in the following, we mainly describe the results of initiated behaviors. Although social rank remained unchanged, we observed a significant main effect of time on aggressive but not submissive behaviors (Table 2), noting that both were used to calculate social rank. Specifically, after chronic stress, the decrease in aggressive behaviors became significant after four weeks of reintroduction to social housing (Observation 3Bvs1: *p* = 0.013) (Figure 6A). For prosocial behaviors (i.e., grooming and proximity), we found a significant main effect of time on proximity but not grooming (Table 2). *Post hoc* tests revealed that, after chronic stress, there was a significant increase in proximity (Observation 3Avs1: iPro: *p* < 0.001), which did not recover after four weeks of social housing (Observation 3Bvs1: iPro: *p* = 0.025; Observation 3Bvs3A: iPro: *p* = 0.882) (Figures 6F and 6H). These findings suggest that prosocial behaviors increased after chronic stress, and such an increase lasted relatively long. Taken together, after chronic stress, although social ranks remained the same, the colonies exhibited a decrease in aggressive behaviors and an increase in prosocial behaviors, indicating more harmonious group relationships.

**Table 2:** Results of two-way repeated measures ANOVAs with Time as the within-subjects factor and Rank as the between-subjects factor for behavioral responses to chronic stress

Behavior	Main Effect of Time			Main Effect of Rank			Interaction		
	F	*p*	Partial η2	F	*p*	Partial η2	F	*p*	Partial η2
iAgg	5.254	0.014^∗^	0.323	6.064	0.032^∗^	0.355	3.139	0.063	0.222
rAgg	3.805	0.038^∗^	0.257	8.250	0.015^∗^	0.429	5.297	0.013^∗^	0.325
iSub	0.315	0.608	0.028	1.620	0.229	0.128	0.315	0.608	0.028
rSub	0.377	0.691	0.033	0.113	0.743	0.010	1.894	0.174	0.147
iGro	2.585	0.126	0.190	8.455	0.014^∗^	0.435	0.139	0.771	0.012
rGro	1.857	0.195	0.144	0.422	0.529	0.037	3.197	0.086	0.225
iPro	10.456	0.007^∗∗^	0.487	0.120	0.736	0.011	0.070	0.814	0.006
rPro	8.877	0.009^∗∗^	0.447	1.044	0.329	0.087	0.346	0.595	0.030

∗*p* < 0.05, ∗∗ *p* < 0.01.

Next, we explored the aforementioned changes at the individual level by examining the relationships between social behaviors and rank. As social rank was calculated based on aggressive and submissive behaviors, we mainly focused on associations of social rank with prosocial behaviors. Before chronic stress (Observation 1), social rank was significantly positively correlated with received grooming but negatively correlated with initiated grooming (Figure 4C, Observation 1 row). However, the relationship between social rank and received grooming vanished after chronic stress (Figure 4C, Observation 3A row) and did not recover after four weeks of reintroduction to social housing (Figure 4C, Observation 3B row), whereas the other relationships found in Observation 1 remained similar (Figure 4C, rPro, iGro, and iPro columns). These findings indicated that chronic stress might lead to changes in the pattern of received grooming in social groups. To further explore changes in received grooming at the individual level, we performed correlation analyses (Figure 4D). Our results revealed significant positive correlations between social rank and changes in received proximity after chronic stress (Figure 4D, rPro – Δ Observation 3Avs1), indicating that higher social rank was associated with higher increases in received proximity. We also found that changes in prosocial behaviors (especially received ones) during four weeks of reintroduction to social housing were negatively correlated with social rank (Figure 4D, Δ Observation 3Bvs3A row): the higher the social rank, the less the increase. These findings provided an explanation for why the relationship between social rank and received grooming did not recover after four weeks (Observation 3B).

**Figure 7:**
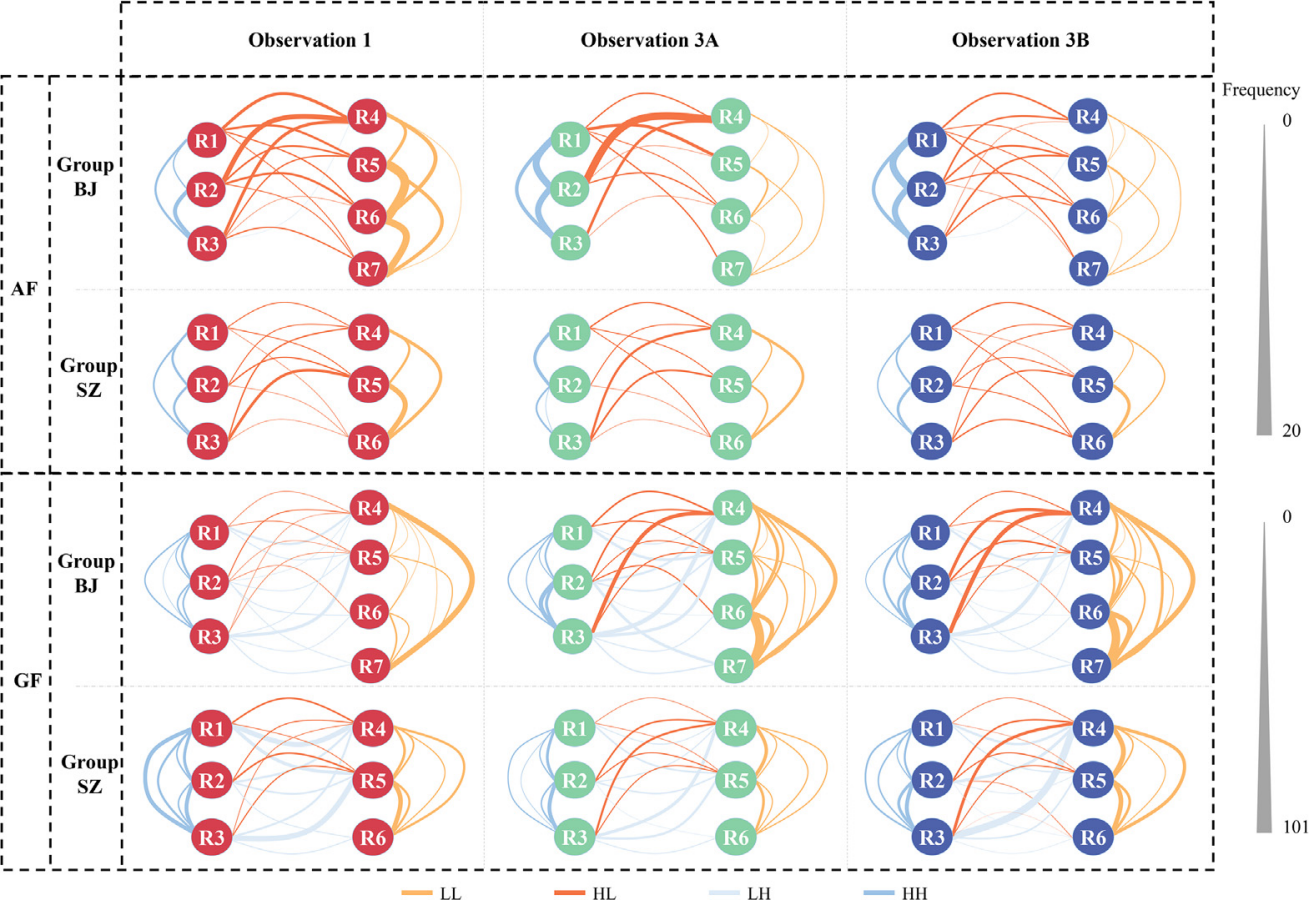
Aggressive behaviors and grooming before and after chronic stress among individuals R1-R7 represent individuals from high to low social rank. Black triangles represent the scale range for all four types of interactions: LL, HL, LH, and HH. Note that, for each kind of social behavior, the scale ranges for all four types of interactions are the same. AF: the frequency of aggressive behaviors, GF: the frequency of grooming.

**Figure 8:**
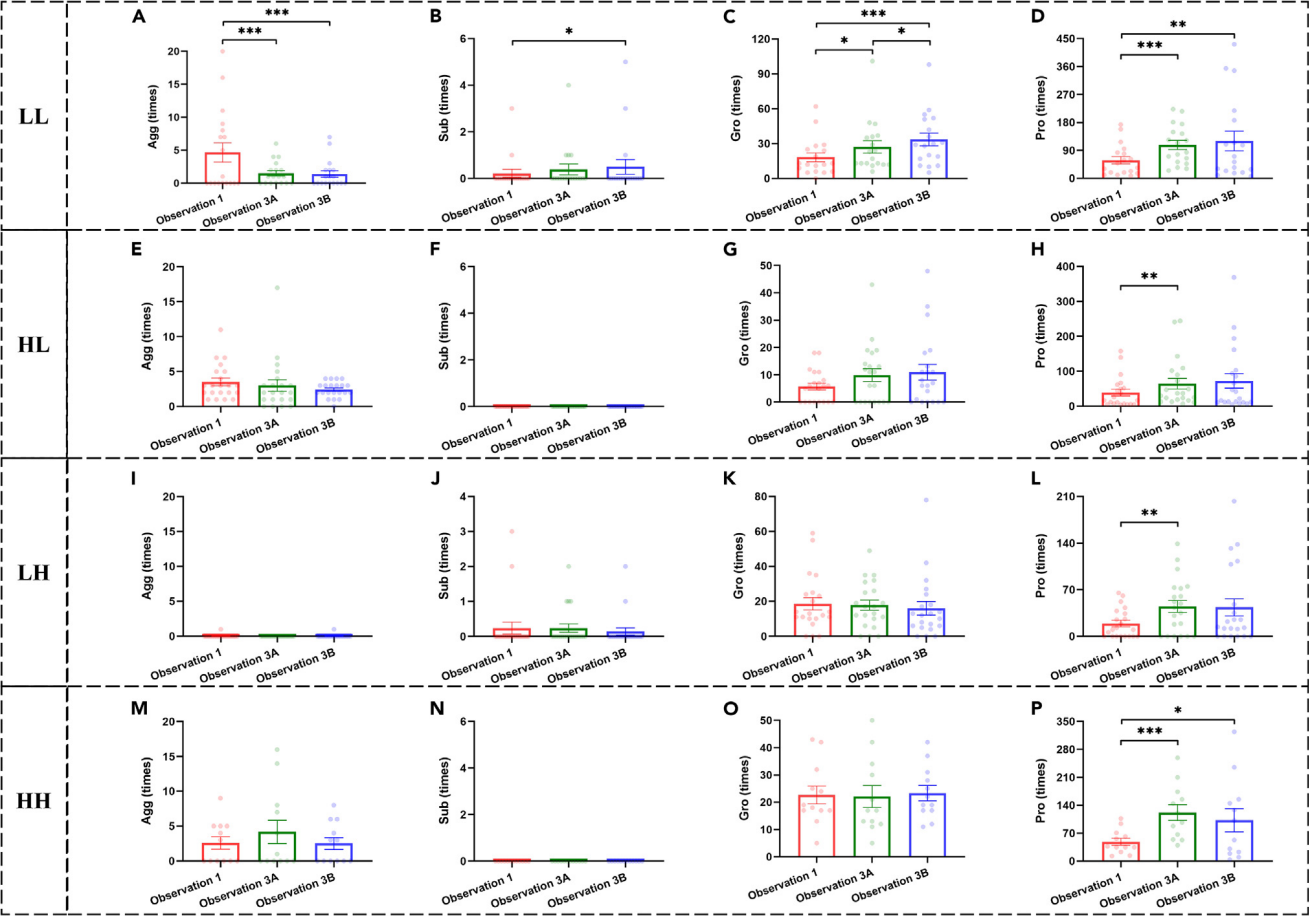
Social behaviors under different types of interactions before and after chronic stress (A–D) show behaviors initiated by low-ranking animals (LR) and received by LR (LL). (E–H) show behaviors initiated by high-ranking animals (HR) and received by LR (HL). (I–L) show behaviors initiated by LR and received by HR (LH). (M–P) show behaviors initiated by HR and received by HR (HH). Black ∗*p* < 0.05, ∗∗*p* < 0.01, ∗∗∗*p* < 0.001 corrected. Error bars indicate standard error. Data points represent the frequencies of each pair (directed social behavior initiated by one monkey and received by another monkey) within each group. For example, in LL, there are 12 data points for each observation, representing the sum of 6 ($A_{3}^{2}$) pairs from directional interactions between every two of the three LR animals within each group. Agg: aggressive behaviors, Sub: submissive behaviors, Gro: grooming, Pro: proximity.

The earlier analyses indicated that lower ranking animals received more prosocial behaviors (e.g., grooming) after chronic stress. To investigate who initiated the increase in prosocial behaviors, we divided each social group into two subgroups (HR and LR) based on social rank (as mentioned earlier) and analyzed four types of interactions: LL (initiated by LR and received by LR), HL (initiated by HR and received by LR), LH (initiated by LR and received by HR), and HH (initiated by HR and received by HR). We also analyzed aggressive and submissive behaviors to provide a comprehensive picture. We found that decreased aggressive behaviors and increased grooming were primarily from LR to LR (Figure 7). To confirm this impression, we conducted two-way repeated ANOVAs with time as the within-subjects factor and type (HH, HL, LH, and LL) as the between-subjects factor (Table 3). We did find significant main effects of time and type on all social behaviors, except for submissive ones. Significant interactions were found in aggressive behaviors and grooming but not in submissive behaviors and proximity. Submissive behaviors remained unchanged after chronic stress and were similar across the different interaction types (Figures 8B–8F, 8J, and 8N). Proximity increased after chronic stress but showed similar changes across interactions (Figures 8D–8H, 8L, and 8P). *Post hoc* tests confirmed that the decrease in aggressive behaviors and the increase in grooming were predominantly from LR to LR (Figures 8A and 8C) (Observation 3Avs1: Agg, *p* < 0.001; Gro, *p* = 0.038; Observation 3Bvs1: Agg, *p* < 0.001; Gro, *p* < 0.001). These findings suggest that the more harmonious group relationships after chronic stress were mainly due to changes in social behaviors among LR animals.

**Table 3:** Results of two-way repeated measures ANOVAs with Time as the within-subjects factor and Type as the between-subjects factor for changes in social interactions induced by chronic stress

Behavior	Main Effect of Time			Main Effect of Type			Interaction		
	F	*p*	Partial η2	F	*p*	Partial η2	F	*p*	Partial η2
Agg	4.791	0.012^∗^	0.066	6.911	<0.001^∗∗∗^	0.234	4.140	0.001^∗∗∗^	0.154
Sub	0.360	0.698	0.005	1.858	0.145	0.076	1.027	0.411	0.043
Gro	3.288	0.050	0.046	6.840	<0.001^∗∗∗^	0.232	2.459	0.038^∗^	0.098
Pro	25.555	<0.001^∗∗∗^	0.273	3.736	0.015^∗^	0.142	1.636	0.175	0.067

∗*p* < 0.05, ∗∗∗ *p* < 0.001.

**Figure 9:**
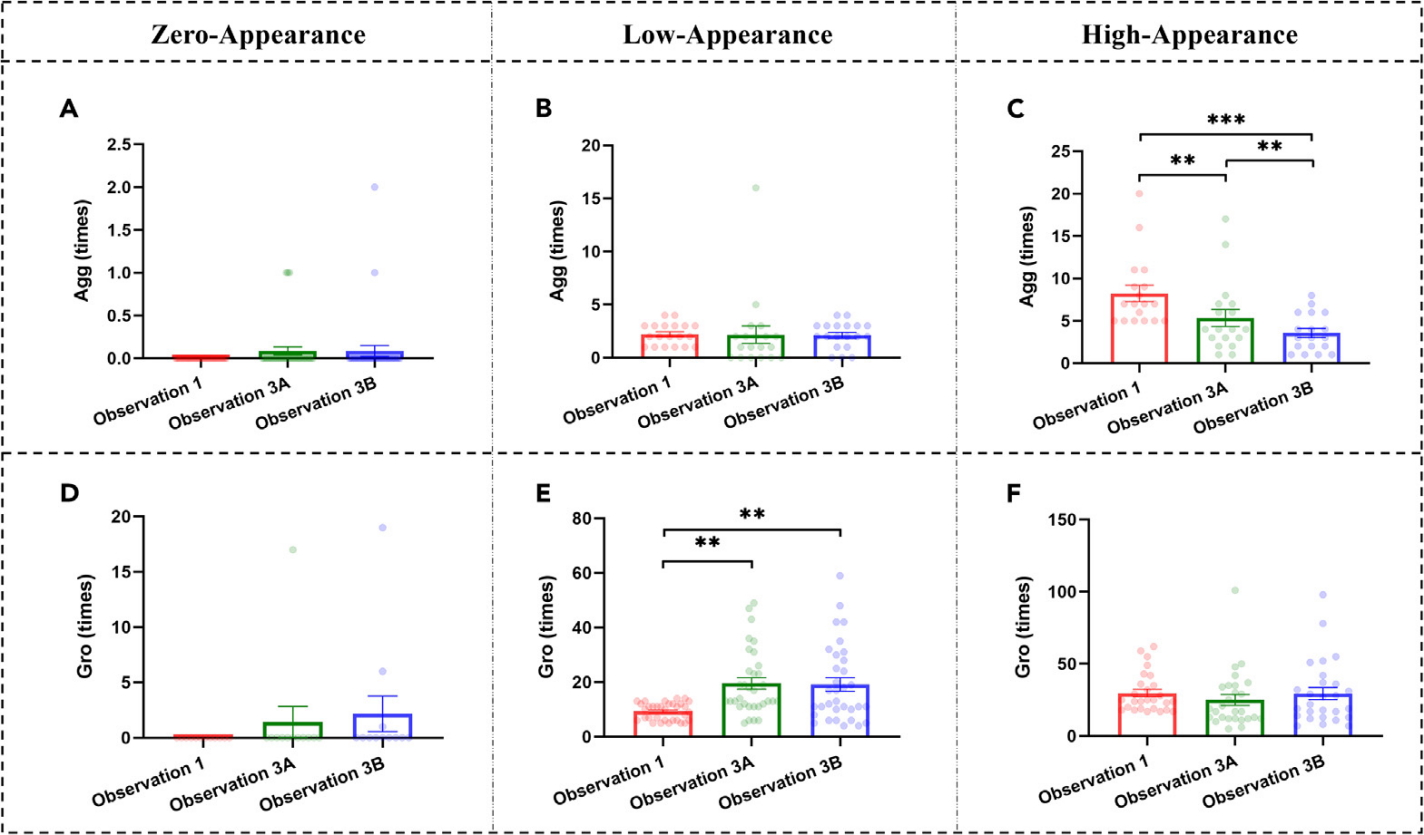
Aggressive behaviors and grooming in pairs with different appearance (A–C) show aggressive behaviors in Zero-Appearance, Low-Appearance, and High-Appearance pairs. (D–F) show grooming in Zero-Appearance, Low-Appearance, and High-Appearance pairs. Black ∗*p* < 0.05, ∗∗*p* < 0.01, ∗∗∗*p* < 0.001 corrected. Data points represent the frequencies of each pair (directed social behavior initiated by one monkey and received by another monkey) within each group. Agg: aggressive behaviors, Gro: grooming.

Furthermore, we investigated whether changes in social interaction occurred in existing pairs, which referred to directed social behavior initiated by one monkey and received by another monkey within each group. Based on the aforementioned findings, we next mainly focused on aggressive behaviors and grooming. For each kind of behavior, based on frequencies observed in Observation 1, we divided the pairs into three subgroups: Zero-Appearance (e.g., no such behavior observed, Agg: *n* = 35; Gro: *n* = 12), Low-Appearance (Agg: frequency >0 and <5, *n* = 19; Gro: frequency >0 and <17, *n* = 33), and High-Appearance (Agg: frequency ≥ 5, *n* = 18; Gro: frequency ≥ 17, *n* = 27). The splitting points for separating Low- and High-Appearance subgroups were selected to maintain a similar sample size for both subgroups. For each pair, we investigated changes in different subgroups across the three observation points (i.e., Observation 1, 3A, and 3B), using two-way ANOVAs with time as the within-subjects factor and appearance as the between-subjects factor (Table 4). We found significant main effects of appearance and interactions between time and appearance for both aggressive behaviors and grooming (Figure 9). A significant main effect of time was found on aggressive behaviors but not grooming. *Post hoc* tests revealed that there were no significant changes in aggressive behaviors of Zero-Appearance and Low-Appearance pairs, whereas aggressive behaviors of High-Appearance pairs decreased significantly after chronic stress and even after the four-week social housing period (Figure 9C; Observation 3Avs1: *p* = 0.002; Observation 3Bvs3A: *p* = 0.005; Observation 3Bvs1: *p* < 0.001). For grooming, there were no significant changes in Zero-Appearance and High-Appearance pairs, whereas grooming of Low-Appearance pairs increased significantly after chronic stress and did not recover after the four-week social housing period (Figure 9E; Observation 3Avs1: *p* = 0.001; Observation 3Bvs3A: *p* = 0.842; Observation 3Bvs1: *p* = 0.004). These findings suggested that changes in social interaction (e.g., decreased aggressive behaviors and increased grooming) induced by chronic stress mainly occurred in existing pairs. Moreover, chronic stress might facilitate low-appearance Gro pairs but inhibit high-appearance Agg pairs.

**Table 4:** Results of two-way repeated measures ANOVAs with Time as the within-subjects factor and Appearance as the between-subjects factor for changes in behavior pairs induced by chronic stress

Behavior	Main Effect of Time			Main Effect of Appearance			Interaction		
	F	*p*	Partial η2	F	*p*	Partial η2	F	*p*	Partial η2
Agg	10.038	<0.001^∗∗∗^	0.127	95.831	<0.001^∗∗∗^	0.735	9.320	<0.001^∗∗∗^	0.213
Gro	1.992	0.149	0.028	27.361	<0.001^∗∗∗^	0.442	3.901	0.009^∗∗^	0.102

∗∗*p* < 0.01, ∗∗∗*p* < 0.001.

We conducted further analyses to investigate such impressions by calculating the correlations between initial values (Observation 1) and changes after chronic stress (Δ Observation 3Avs1). Since there were no significant changes in Zero-Appearance pairs for both aggressive behaviors and grooming, we next focused on the existing pairs. That is, non-existing pairs were excluded in the following correlation analyses. We found significant negative relationships between Observation 1 and Δ Observation 3Avs1 for both aggressive behaviors (r = −0.602, *p* < 0.001) and grooming (r = −0.540, *p* < 0.001). These findings confirmed that, after chronic stress, the higher the appearance of the Agg pairs, the more they were inhibited, whereas, the higher the appearance of the Gro pairs, the more they were facilitated.

### Relationships between HC and OT/AVP as well as prosocial behaviors

Previous studies have reported that OT/AVP and social support (mainly from prosocial behaviors) may be involved in stress regulation.^37^ To further explore the mechanisms underlying coping stress, we examined the associations among changes in HC, OT/AVP, and prosocial behaviors after chronic stress.

Prior to chronic stress exposure, we did not observe any relationship between HC and OT (r = 0.035, *p* = 0.911), between HC and AVP (r = 0.048, *p* = 0.876), or between HC and prosocial behaviors (Figure 4E, HC row). However, we did find significant relationships between received grooming and OT, as reported in previous studies (Figure 4E, OT row).^39^^,^^40^

Next, we examined the associations among changes caused by chronic stress (e.g., ΔHC 2vs1). Our results revealed no significant relationship between changes in HC and OT (r = −0.098, *p* = 0.749) or between changes in HC and AVP (r = 0.300, *p* = 0.320). Note that, after chronic stress, Sample 2 was collected before animals were returned to normal social housing, that is, before Observation 3A. Therefore, for one monkey, levels of hormones in Sample 2 may be related to its initiated behaviors but not received behaviors in Observation 3A. Therefore, we mainly examined the correlations between changes in hormones and initiated behaviors, and no significant relationships were found (Table S1).

Finally, we investigated changes in Phase 3 (e.g., Δ HC 3vs2). We did not observe a significant relationship between changes in HC and AVP (r = 0.534, *p* = 0.060) or between changes in HC and OT (r = 0.047, *p* = 0.880). Again, no significant relationships were found between changes in HC and prosocial behaviors (Figure 4F, Δ HC 3vs2 row). Of note, changes in OT/AVP during four weeks of reintroduction to social housing were positively correlated with changes in frequencies of prosocial behaviors (Figure 4F, Δ AVP 3vs2 and Δ OT 3vs2 rows). During four weeks of reintroduction to social housing, significant relationships between changes in initiated and received prosocial behaviors were also found (Δ Observation 3Bvs3A: for Gro, r = 0.604, *p* = 0.029; for Pro, r = 0.872, *p* < 0.001). Note that such relationships vanished or weakened when controlling for changes in OT levels (for Gro, r = 0.337, *p* = 0.384; for Pro, r = 0.609, *p* = 0.036) or AVP levels (for Gro, r = 0.498, *p* = 0.099; for Pro, r = 0.681, *p* = 0.015), indicating social behaviors, especially grooming, may be deeply related to hormones (e.g., OT).

As the decrease in HC in Phase 3 mainly occurred in HR animals, we again divided the social group into two subgroups (HR and LR) and reanalyzed the associations among changes in HC, OT/AVP, and prosocial behaviors. No matter whether in HR or LR animals, no significant relationship was found between HC and AVP (HR: r = −0.637, *p* = 0.174; LR: r = 0.727, *p* = 0.064) or prosocial behaviors (Figure S2). However, our results showed a significant negative correlation in HR animals but not in LR ones between changes in HC (ΔHC 3vs2) and OT (ΔOT 3vs2) (HR: r = −0.823, *p* = 0.044; LR: r = 0.254, *p* = 0.583) (Figure S2C). That is, the increase in OT was associated with the decrease in HC in HR animals. Our findings suggested that the increase (albeit limited) in OT levels might be associated with the reduced stress in HR animals.

## Discussion

In the present study, we investigated stress levels and physiological and behavioral responses of monkeys in two colonies to chronic stress primarily induced by social isolation. Our results showed that, following exposure to chronic stress, 1) HR animals exhibited higher stress levels than LR animals, 2) social hierarchies persisted, with notable improvements in group harmony, and 3) changes were observed in both physiological and behavioral responses, which might be influenced by social rank.

### Changes in HC levels associated with social hierarchy

After chronic stress, we observed a significant increase in HC levels, which remained elevated even after four weeks of returning to normal social housing. Notably, changes in HC levels were significantly associated with social rank, with higher ranking individuals exhibiting a more pronounced increase in HC levels. These findings indicate that, when exposed to the same external stress, dominant individuals may be more susceptible to chronic stress than their subordinate counterparts. Similar results have been reported under naturally occurring chronic stress. For example, HR male monkeys excrete higher glucocorticoid levels than LR males under high reproductive pressure.^12^^,^^41^ Notably, these findings may be influenced by confounding factors (e.g., sex hormones) other than chronic stress. To control for potential confounding effects on stress responses arising from reproductive seasons on levels of sex hormones in previous studies, we conducted our experiments during the non-reproductive season. To account for possible fluctuations in sex hormones during the ovulatory menstrual cycle and their influence on cortisol levels, we employed HC sampling, which provides an assessment of cortisol levels over a period (e.g., isolation period in the present study). Furthermore, we implemented a four-week isolation period, which aligns with the duration of an entire ovulatory menstrual cycle. As such, normal fluctuations in sex hormones throughout experiments may not fully explain our results. Our study provides evidence that HR primates exhibit greater stress responses to chronic stress than their LR counterparts. Note that a relationship between female social hierarchy and sex hormones has been identified.^42^^,^^43^ Moreover, previous studies, primarily conducted in rodents, have established a relationship between sex hormones and stress responses (for review, see Væroy et al.^44^). Regrettably, sex hormone levels were not monitored in the present study. Future studies can explore the variations in sex hormone changes among animals with different social ranks exposed to chronic stress and even employ exogenous sex hormone manipulations. Such studies may provide valuable insights into the underlying mechanisms of the observed relationship between social rank and responses to chronic stress, which may be mediated by sex hormones.

### Changes in immune responses associated with social hierarchy

In the present study, we utilized a mild chronic stress protocol, wherein all members of the social groups were co-housed in the same room, with restricted visual and tactile contact but allowed for olfactory and auditory interactions. This mild chronic stress paradigm may account for the observation that the increase in IgG/IgA levels was not immediately evident following isolation but initiated from Week 2 onwards. Note that fecal samples were collected on a weekly basis. Consequently, the increase in IgG/IgA levels might have occurred between Week 1 and Week 2.

Notably, during chronic stress, we observed less pronounced immune responses (i.e., the increase in IgG levels in Week 3) in dominant individuals who were more susceptible to chronic stress (Figure S1). There could be two possible explanations for this phenomenon. Firstly, the relationship between stress-induced immune responses and stress levels may be complex and may not simply follow a linear positive correlation. Furthermore, social rank may be another potential factor that can influence immune responses to chronic stress in addition to stress levels. As such, we did not observe more pronounced immune responses in animals with higher levels of HC. Alternatively, a previous study has suggested that IgG may modulate adrenocorticotropic hormone (ACTH)-induced cortisol secretion, indicating the involvement of IgG in stress regulation.^45^ Then, the less increased IgG levels in HR animals may indicate less effective stress regulation, potentially contributing to higher HC levels in these animals. Further investigations are needed to explore these possibilities comprehensively.

### Retained social structure but with more harmonious group relationships after chronic stress

In previous studies, subjects were selected from different colonies and then returned to their original social groups after chronic stress.^29^^,^^32^ Therefore, it is difficult to assess the impact of chronic stress on social structure and relationships as not all group members experienced stress. In one research, entire social groups were subjected to single housing but were allowed visual contact and could direct aggressive and submissive behaviors toward others.^27^ As such, the reported unaffected social hierarchies may be due to the persistence of such social behaviors, which are major determinants of social hierarchies.^30^ In the present study, all social group members were exposed to the same chronic stress that limited visual and tactile contact, while still enabling olfactory and auditory contact. This approach allowed us to investigate the effects of chronic stress on group-level responses with limited confounding effects of social behavior. Our study provides clear and convincing evidence that social hierarchy remains unchanged when exposed to the same chronic stress conditions.

Importantly, our study demonstrated that, despite the social structure remaining unchanged, colonies exposed to chronic stress exhibited increased prosocial behaviors and decreased aggressive behaviors, consistent with previous studies.^27^^,^^32^ However, previous studies did not^27^ or could not^32^ investigate the potential relationship between changes in behaviors and social rank and were thus unable to determine whether the entire social group or only specific individuals were responsible for the observed changes. In this study, we investigated changes in social behavior in relation to social rank and recorded both initiators and receivers of behaviors to gain a comprehensive understanding of changes at both the individual and group level. Our results revealed a significant negative correlation between social rank and changes in received grooming during four weeks of reintroduction to social housing after chronic stress (Δ Observation 3Bvs3A), with HR individuals showing a lower increase in received grooming and LR individuals showing a higher increase in received grooming. Further analyses found that LR monkeys exhibited decreased aggressive behaviors but increased grooming toward other LR individuals after chronic stress exposure (Figures 7 and 8). Note that we also observed significant changes in proximity across all four types of interactions (i.e., LL, HL, LH, and HH), encompassing the entire colony. Our findings suggest the development of a more harmonious group after chronic stress, with notable contributions from LR animals. The role of prosocial behaviors in stress regulation has been extensively documented in previous studies.^46^^,^^47^^,^^48^ Although LR animals were less affected by chronic stress, they still engaged in more changes in prosocial behaviors as compared to HR ones, which may serve as a stress-coping mechanism and facilitate a more efficient recovery from chronic stress for these animals.

Notably, LR monkeys received friendly behaviors mainly from other LR individuals in natural social environments. Previous studies have demonstrated that prosocial behavior can establish and strengthen social bonding between animals and console distressed conspecifics by providing comfort to them (e.g., grooming).^46^ In the present study, we found that increased grooming induced by chronic stress mainly occurred within existing pairs before chronic stress. For aggressive behaviors, the decrease could only happen within existing pairs. Note that no significant changes were found in Zero-Appearance pairs following exposure to chronic stress or even upon reintroduction of social housing. Therefore, our findings indicated that, after chronic stress, animals, especially LR ones, reinforced existing bonds. This finding is partly in line with previous research, which showed that, under naturally occurring chronic stress (i.e., hurricane disaster), monkeys, especially those at the edge of social networks, increased social bonding.^47^ However, in that study, monkeys established new social connections rather than strengthening existing partnerships to improve social bonding. The difference from our findings may be due to uncontrolled variabilities, such as missing partners and food shortages. In the current study, social group size and composition were consistent before and after chronic stress exposure, and adequate food and water were provided. Previous studies have reported that interacting with bond partners, such as through grooming, can reduce stress in chimpanzees.^49^ Furthermore, after chronic stress, we observed a decrease in aggressive behaviors mainly within pairs exhibiting high appearance previously, whereas grooming behaviors showed an increase primarily within pairs with low (but not zero) appearance previously. Therefore, our results indicate that, following exposure to chronic stress, individuals’ strategies for coping with chronic stress are to improve existing worse/less close bonds to strengthen social support: they offer more prosocial behaviors (e.g., grooming) toward individuals with whom they have previously shown less friendliness and reduce aggressive behaviors directed toward individuals with whom they have previously initiated more aggressive behaviors.

### Potential mechanisms underlying stress coping through the OT system

After chronic stress (Sample 2), our results showed an increase in levels of OT/AVP and HC. Given the absence of major social behaviors, such as visual or tactile contact during social isolation, the observed increase in OT/AVP levels might be attributable to physiological responses to chronic stress and responsible for increases in HC levels. Previous studies in rodents have provided evidence of the crucial role of OT and AVP in regulating stress-induced hypothalamic-pituitary-adrenal (HPA) axis activity. For instance, OT released from the paraventricular nucleus (PVN) functions as an anxiolytic suppressing HPA axis activity.^50^ Conversely, the PVN of the hypothalamus releases AVP in response to stress, initiating a signaling cascade that results in increased circulating corticosterone levels and the development of psychological and behavioral pathologies.^51^ That is, OT buffers responses to stress, whereas AVP promotes responses to stress.^52^ Indeed, we found that changes in AVP were more similar to those in HC, whereas changes in OT occurred in the opposite direction (e.g., Figure 3).

Considering the anti-stress effect of OT (e.g., reducing cortisol levels),^52^ the observed differential changes in OT between dominant and subordinate individuals following chronic stress may contribute to rank-dependent differences in stress levels after chronic stress (e.g., HC levels). Rodent studies have reported that social status in social hierarchies is linked to individual variations in OT receptor binding densities.^53^^,^^54^ For example, dominant mice have higher OT receptor binding in the nucleus accumbens (NAc) than subordinate ones.^54^ While these variations in OT receptors may not fully explain rank-dependent changes in OT levels after chronic stress found in our study, they indicate potential divergences in the functioning of the OT system between HR and LR animals. Future studies can be designed to directly examine the response of OT neurons to stressful stimuli associated with social status.

In addition to its role in stress buffering, OT also contributes to social behavior regulation across species.^55^^,^^56^ Especially, OT may promote prosocial behaviors. For example, nebulized OT can increase the frequency of grooming during free interactions in female bonobos.^40^ Importantly, increased prosocial behaviors may, in turn, cause an increase in OT, which may explain the continued rise in OT levels even after four weeks of social housing. It is well established that an individual’s OT levels can be influenced by received prosocial behaviors, particularly from bond partners. For example, studies in chimpanzees have shown that OT levels increase after experiencing grooming interactions with bond partners but not with non-bond partners.^39^ Studies in rodents have demonstrated that social touch can promote communication between females by activating parvocellular OT neurons.^57^ In the present study, our data did reveal significant correlations between the increase in OT (ΔOT 3vs2) and in received prosocial behaviors (i.e., ΔrGro 3Bvs3A and ΔrPro 3Bvs3A) during four weeks of reintroduction to social housing.

Crockford et al. (2013) proposed a positive feedback loop through OT to support social bonding.^39^ In the present study, we found significant correlations between changes in initiated and received prosocial behaviors (e.g., grooming) during four weeks of reintroduction to social housing. Such relationships weakened or vanished when controlling for changes in OT levels. Our study suggests that there may be a positive feedback loop between OT and prosocial behaviors after chronic stress, which may be one explanation for the continued rise in OT levels and prosocial behaviors even after four weeks of social housing. First, OT levels increase in response to chronic stress to directly mitigate stress responses (e.g., the increased OT in Sample 2). Second, when social interactions are possible (e.g., in Phase 3), elevated OT levels elicit more initiated prosocial behaviors, thereby reinforcing social bonding, which, in turn, leads to an increase in received prosocial behaviors, which further facilitates OT release. Previous studies have implied that the interactions between social support and OT play a crucial role in suppressing cortisol and subjective responses to psychosocial stress.^48^ Therefore, our results indicate that the positive feedback loop between OT and prosocial behaviors after chronic stress may play an important role in ameliorating the negative impact of stress.

Our study identified an association between changes in OT and HC in HR animals but not in LR ones during four weeks of reintroduction to social housing (Figure S2C), although, in HR animals, the increase in OT during Phase 3 was not significant. In LR animals, although OT levels increased more, changes in HC during Phase 3 were not significant. There are several potential explanations for these observations. One possibility is that the increase in HC in LR animals was comparatively less pronounced than that in HR animals. Consequently, the decrease in HC during the four-week reintroduction to social housing was not significant, and the effects of OT on buffering stress were not apparent. Another possibility is that the buffering effects of OT may be more effective in HR animals than in their LR counterparts. As such, even a subtle increase in OT levels may potentially reduce stress in HR animals. Further studies are needed to investigate these two possibilities.

### Limitations of the study

During Phase 1, we only conducted one observation instead of two observations with a 4-week interval, as we did in Phase 3. In future studies, it would be interesting to conduct such an additional investigation in Phase 1 and subsequently compare it with Observation 1, which would provide more informative insights for interpreting changes during the four weeks of reintroduction to social housing.

In the present study, two colonies were included. Most of the physiological and behavioral responses investigated (as shown in Figures S3–S6) remained similar between the two groups. However, subtle differences were detected. For example, after four weeks of social housing, Group BJ displayed a recovery pattern in AVP levels, whereas Group SZ did not show recovery in such hormonal responses. Note that Group BJ exhibited a higher frequency of aggressive behaviors compared to Group SZ before chronic stress. Therefore, the differences in social environments between the two groups may have influenced their responses to chronic stress (e.g., AVP, which AVP plays a pivotal role in the regulation of aggressive behaviors^58^). Due to the limited sample size (*n* = 7 in Group BJ and *n* = 6 in Group SZ), conducting a meaningful comparison between the two groups and obtaining statistically significant results posed challenges. Therefore, further investigation into the effects of social environments on responses to chronic stress may be necessary in future studies.

### Conclusions

In the present study, we evaluated whether social hierarchy status, a major component of primate social environments, influences stress levels induced primarily by social isolation as well as physiological and behavioral responses to chronic stress. We found that dominant individuals were more susceptible to chronic stress than their subordinate counterparts. Additionally, position in the social hierarchy significantly predicted multiple OT/AVP and behavioral outcomes following chronic stress exposure. Our results indicate that a potential positive feedback loop between OT and social support may play a critical role in regulating responses to stress. Moreover, the effects of OT on buffering stress were more pronounced in HR animals. In addition, our results suggested that, despite the maintenance of social hierarchies, the colonies exhibited more harmonious group relationships after chronic stress. Our study demonstrates the importance of social factors in coping with chronic stress and provides insight into how individual differences shape stress response, which could facilitate the development of tailored and personalized therapies for coping with chronic stress and, by extension, stress-related disorders.

## STAR★Methods

### Key resources table

**Table:** 

REAGENT or RESOURCE	SOURCE	IDENTIFIER
**Biological samples**		

Healthy macaque blood and hair samples	Beijing Prima Biotech Inc., Beijing, China	N/A
Healthy macaque blood and hair samples	Hubei Topgene Biotechnology Co., Ltd., Suizhou, China	N/A

**Critical commercial assays**		

Oxytocin ELISA Kit	ENZO	ADI-900-153A-0001
Cortisol ELISA Kit	ENZO	ADI-900-071
Arg8-Vasopressin ELISA Kit	ENZO	ADI-900-017A

**Experimental models: Organisms/strains**		

Rhesus macaques (Macaca mulatta)	Beijing Prima Biotech Inc., Beijing, China	N/A
Rhesus macaques (Macaca mulatta)	Hubei Topgene Biotechnology Co., Ltd., Suizhou, China	N/A

**Software and algorithms**		

SPSS v26	SPSS Inc., Chicago, IL, USA	https://www.ibm.com/cn-zh/spss
JASP	University of Amsterdam	https://jasp-stats.org/
MATLAB (v.2020a)	MathWorks	https://www.mathworks.com/
GraphPad Prism	GraphPad Software	https://www.graphpad.com/

### Resource availability

#### Lead contact

Further information and requests should be directed to and will be fulfilled by the lead contact, Ning Liu (liuning@ibp.ac.cn).

#### Materials availability

This study did not generate new unique reagents.

#### Data and code availability

• The data supporting the conclusions of this manuscript has been deposited on Zenodo: https://doi.org/10.5281/zenodo.10906905, and are publicly available as of the date of publication.
• This paper does not report original code.
• Any additional information required to reanalyze the data reported in this paper is available from the lead contact upon request.

### Experimental model and study participant details

Due to ethical concerns, we endeavored to conduct this experiment with a substantial number of animals to accommodate statistical analyses while minimizing the use of animals. Two groups of adult female macaques (*Macaca mulatta*) were used in the current study, named Group BJ *(n* = 7, Beijing Prima Biotech Inc., Beijing, China) and Group SZ (*n* = 6, Hubei Topgene Biotechnology Co., Ltd., Suizhou, China). Note that we analyzed data from all animals (n = 13) within these groups. These monkeys were four years old and had been group housed together in the same colony for two years before the start of the present study. All experimental procedures complied with the US National Institutes of Health Guide for the Care and Use of Laboratory Animals and were approved by the Institutional Animal Care and Use Committee of the Institute of Biophysics, Chinese Academy of Sciences (CAS). Expert technicians performed all experimental procedures, with efforts made to minimize any potential suffering. The housing room was maintained on a standard light/dark (12/12 h) cycle. All monkeys were provided with monkey chow biscuits, fresh fruit, and vegetables twice daily and had unlimited water access during the entire experiment.

### Method details

#### Experimental design

The experiment comprised three phases (Figure 1). In Phase 1, the monkeys were housed in their original home cages (6.0 × 3.5 × 3.0 m (L×W×H)). At the onset of Phase 2, all animals were transferred to single cages (0.8 × 0.9 × 1.0 m (L×W×H)) within the same room. To minimize the suffering caused by social isolation while maintaining experimental integrity, Phase 2 lasted four weeks compared to two to three months or even longer in previous studies.^27^^,^^29^^,^^32^ Animals from the same group were kept in the same room. Subjects remained in olfactory and auditory but not visual or tactile contact with each other. Moreover, to successfully induce chronic stress in a shorter period compared to previous studies, two stressors were applied alongside social isolation to simulate unpredictable stress conditions: space restriction and intimidation.^59^ Space restriction was conducted by reducing the space of the cage with the push-pull device equipped on the cage, and intimidation was implemented by an operator waving a capture net in front of the cage. These two stressors were randomly carried out once and lasted 10–15 min per day. They could be given consecutively in the morning or afternoon with randomized intervals or separately, with one stressor in the morning and the other in the afternoon. In Phase 3, all monkeys were returned to their original home cages.

#### Behavioral data collection and analysis

Digital cameras were fixed in front of the home cages to record animal behaviors. To avoid disturbing the animals, no observers remained near the cages except for regular and necessary processes (e.g., feeding and cleaning).

During Phase 1, three-hour behavioral recordings were collected for each monkey twice a day for one week (Observation 1): once in the morning (08:00−11:00) and once in the afternoon (14:00−17:00). In Phase 3, behavioral data were collected in week 1 (Observation 3A) and week 4 (Observation 3B) using the same procedures as in Observation 1 (Figure 1). Data from one day of Group BJ in Observation 3B was missing due to a technological issue. Therefore, to ensure data consistency and quality, data were summarized across recordings of six consecutive days within a week for all three observations. That is, the dataset for all three observation points was derived from 36-hour video recordings over six consecutive days.

For this study, we focused on four behaviors: aggressive behaviors, submissive behaviors, proximity, and grooming. Behaviors in the home cages were classified into these types using the “posture-location-action” rule. Aggressive and submissive behaviors were used to calculate the social rank of each monkey in the group according to David’s score (DS).^30^ Aggressive behaviors included bite, slap, grab, stare threat, open-mouth threat, chase, and displacement, while submissive behaviors included scream threat, crouch, fleeing, lip smack, grimace, submissive present, and moving away. In addition, two prosocial behaviors were also measured: grooming and proximity. The frequencies of each behavior as well as the corresponding initiators and receivers were recorded and analyzed.

#### Sample collection and measurement

Blood and hair samples as well as weights were collected at the end of each phase, corresponding to samples 1, 2, and 3, respectively. Sample collection, storage, and processing were the same as described in previous research.^59^ In brief, expert technicians manually restrained each monkey and drew 3 mL of blood from the femoral vein into ethylenediaminetetraacetic acid (EDTA) vacuum tubes. Hair from the back of the monkey’s neck was shaved and placed into a small aluminum foil pouch to avoid light. Following collection, blood tubes were centrifuged at 4°C, 8000 r/min for 10 minutes, and the resulting plasma samples were stored at -20°C until assayed. The plasma concentrations of OT and AVP, as well as the concentration of HC, were quantified using commercially available enzyme-linked immunosorbent assays (ELISA) (Enzo Life Sciences, Farmingdale, NY, OT: ADI-900-153A-0001, AVP: ADI-900-071; Cortisol: ADI-900-017A). Assays were conducted in accordance with the manufacturer’s protocols.

To evaluate and monitor responses induced by social isolation without disturbing the animals, fecal samples were collected since the animals were transferred into single cages and at weekly intervals in Phase 2, resulting in a total of five samples per monkey (Figure 1), which were stored at -20°C until assayed. We obtained sufficient fecal samples from all monkeys at all time points except one monkey on one day, which was replaced by a fecal sample collected the day after. As stress can lead to increased immune responses,^35^^,^^36^ we quantified the fecal concentrations of two major immunoglobulins (IgG and IgA) using commercially available ELISA (Enzo Life Sciences, Farmingdale, NY).

Due to the limited sample size, we did not conduct duplicate testing. The inter-assay coefficients of variation (CVs) based on standard samples were consistently below 9% across all tested concentrations, within the acceptable range [CV<15%]. That is, the assay was considered suitable in terms of accuracy and precision. Note that the experimenter was blind to the samples’ experimental condition (e.g., the animal’s rank, the sample’s phase) at the time of assay to ensure impartiality and minimize potential bias during the assay.

### Quantification and statistical analysis

Data analyses were conducted using SPSS v26 (SPSS Inc., Chicago, IL, USA), JASP (v0.15), and MATLAB (v.2020a). We collapsed data from all animals within two groups, resulting in a sample size of 13 for statistical analyses. To explore changes over time, we treated Time (*n* = 3 for weights, blood and hair samples, and behavioral data; *n* = 5 for fecal samples) as the within-subjects factor. To assess potential differences between high- and low-ranking subjects, we divided each social group into two subgroups based on social rank: high-ranking (HR, DS > 0.5) and low-ranking (LR, DS < 0.5). Then, we performed two-way repeated measures ANOVAs with Time as the within-subjects factor and Type (HR and LR) or Interaction (LL: initiated by LR and received by LR; HL: initiated by HR and received by LR; LH: initiated by LR and received by HR; HH: initiated by HR and received by HR) as the between-subjects factor. The Greenhouse-Geisser correction was used for deviations from Mauchly’s test for sphericity when required. We then followed up with *post hoc* tests, with adjustments for multiple testing using the Holm-Bonferroni method. All *p-*values were corrected unless specified otherwise.

Changes in social behaviors and physiological indicators after chronic stress were calculated using Equation 2:

(Equation 2) $$\Delta X\;ivsj=\frac{\left(X_{i}-\;X_{j}\right)}{X_{j}}$$

where X is the observed variable, i and j are compared sample points.

To assess the relationships among social rank, immune responses (IgG and IgA), hormones (HC, OT, and AVP), and social behaviors, we conducted correlation analyses. Customized non-parametric correlations were performed to compensate for group-level differences: the data were first ranked within each group and then collapsed together; next, two-tailed Pearson correlations were calculated. To evaluate the relationships between changes in behaviors and hormones, we calculated two-tailed Pearson correlations.

We also conducted analyses for each group and obtained similar results (see details in Figures S3–S6).
