# Supplementary material for: Social status predicts physiological and behavioral responses to chronic stress in rhesus monkeys
Source: iScience. 2024 May 21;27(6):110073. doi: 10.1016/j.isci.2024.110073 (PMC11176666; doi:10.1016/j.isci.2024.110073)
Supplement: Document S1. Figures S1–S6 and Table S1 [file mmc1.pdf]

iScience, Volume *27*

Supplemental information

Social status predicts physiological and behavioral responses to chronic stress in rhesus monkeys

Zhiyi Zhang, Xueda Dong, Zhiqiang Liu, and Ning Liu

Supplementary Table

Table S1. Relationships between changes in hormones and initiated prosocial behaviors after chronic stress (related to Figure 4).

	**Δ HC 2vs1**	**Δ AVP 2vs1**	**Δ OT 2vs1**
**Δ iGro 3Avs1**	-0.229(0.452)	0.319(0.288)	-0.396(0.181)
**Δ iPro 3Avs1**	0.019(0.950)	-0.121(0.694)	-0.286(0.343)

Supplementary Figure

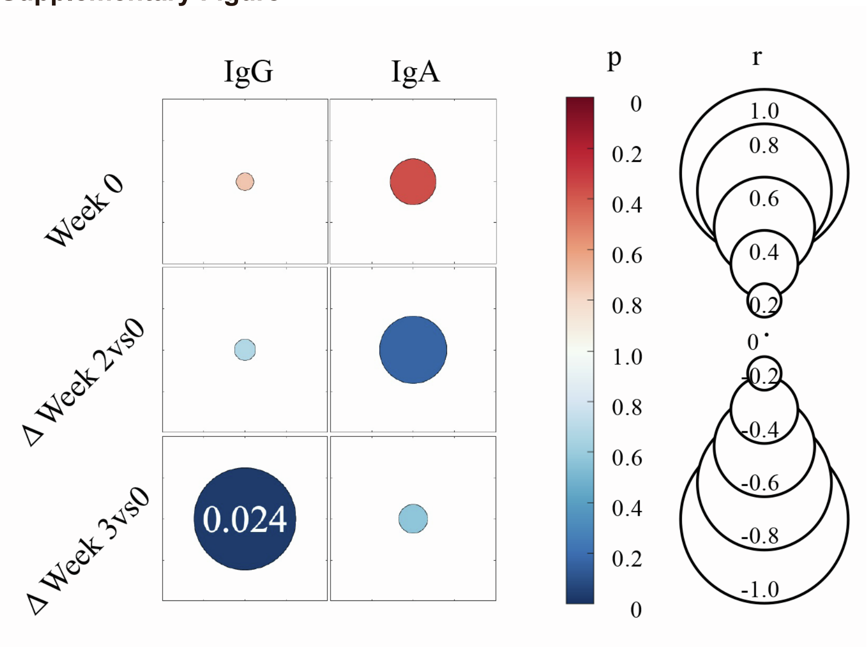

Figure S1. Relationships between social rank and immune responses (related to Figure 4). A red circle indicates a positive correlation coefficient and a blue circle indicates a negative one. The color intensity of the circle indicates the p value (two-tailed). If the p value is less than 0.05, the p value is added to the circle. A full circle corresponds to r = 1 or -1 and an empty circle corresponds to r = 0.

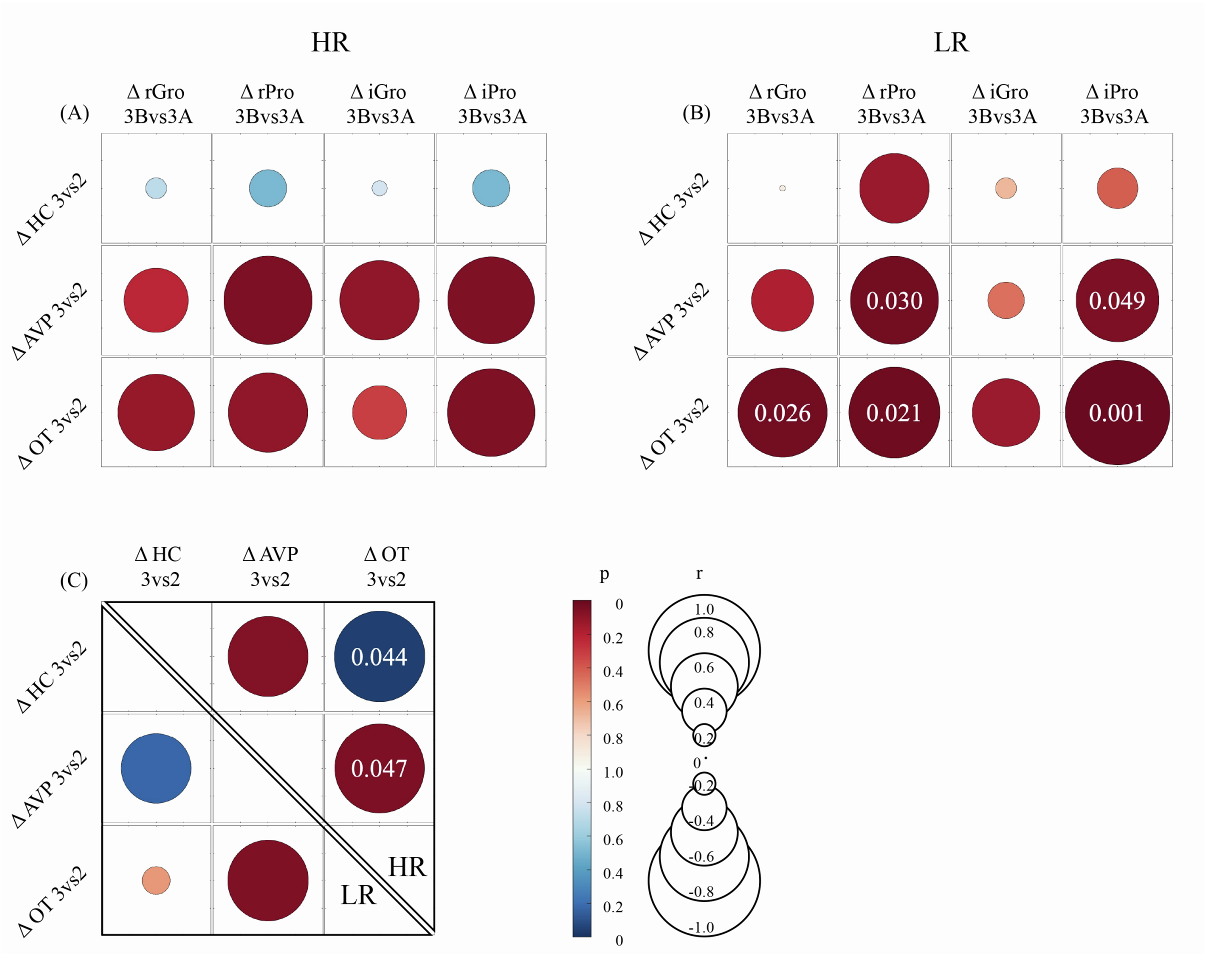

Figure S2. Relationships among changes in hormones and prosocial behaviors in Phase 3 (related to Figure 4).

(A) and (B) show relationships between changes in hormones and changes in prosocial behaviors during four weeks of reintroduction to social housing in high-ranking and low-ranking animals, respectively. (C) shows relationships among changes in hormones during four weeks of reintroduction to social housing in high-ranking (upper triangle) and low-ranking animals (lower triangle). A red circle indicates a positive correlation coefficient and a blue circle indicates a negative one. The color intensity of the circle indicates the p value (two-tailed). If the p value is less than 0.05, the p value is added to the circle. A full circle corresponds to r = 1 or -1 and an empty circle corresponds to r = 0. Gro: grooming, Pro: proximity.

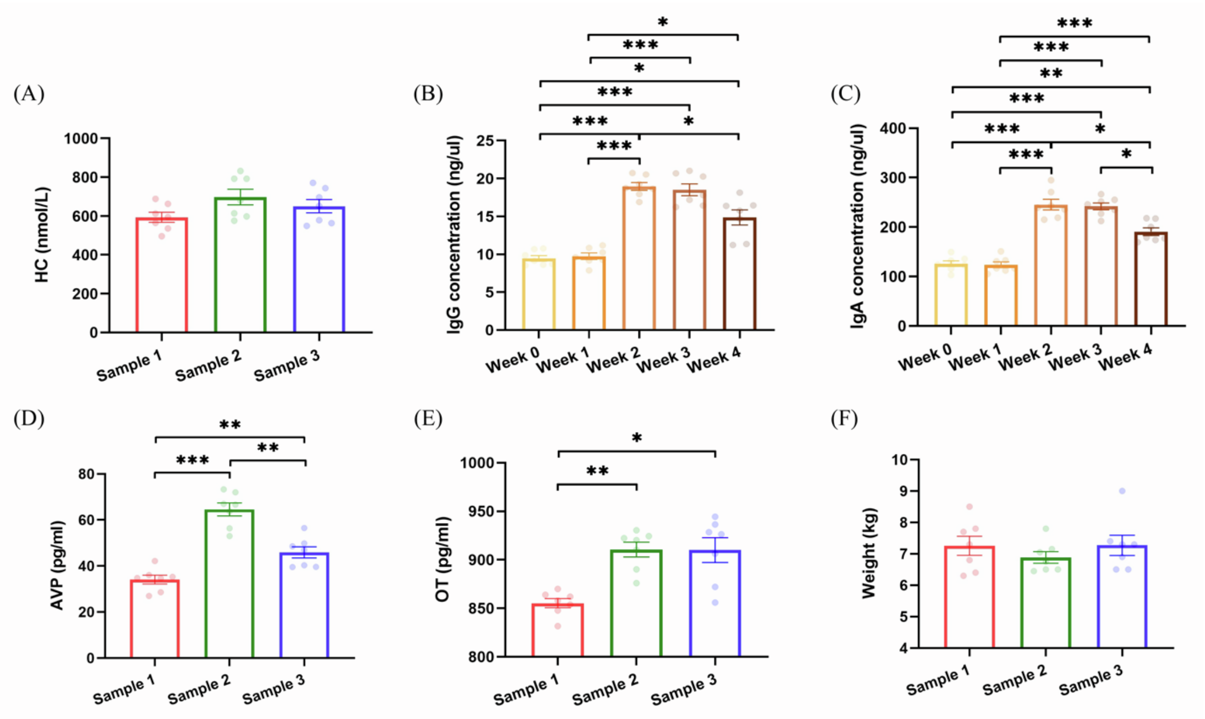

Figure S3. Physiological levels before, during, and after chronic stress in Group BJ (related to Figure 3).
(A) shows HC levels before and after chronic stress. (B) and (C) show IgG and IgA levels during chronic stress. (D)-(F) show AVP, OT, and weight levels before and after chronic stress, respectively. Black * $p < 0.05$, ** $p < 0.01$, *** $p < 0.001$ corrected. Error bars indicate standard error. Individual data points are shown in corresponding colors.

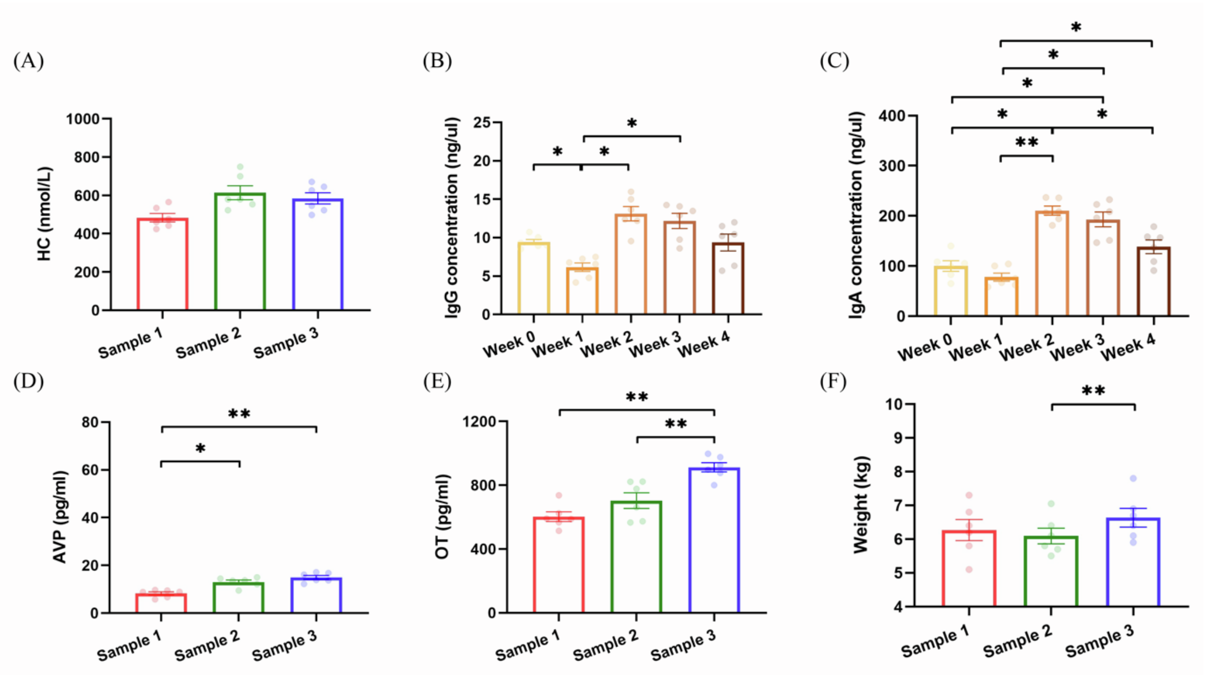

Figure S4. Physiological levels before, during, and after chronic stress in Group SZ (related to Figure 3).

(A) shows HC levels before and after chronic stress. (B) and (C) show IgG and IgA levels during chronic stress. (D)-(F) show AVP, OT, and weight levels before and after chronic stress, respectively. Black * $p < 0.05$, ** $p < 0.01$ corrected. Error bars indicate standard error. Individual data points are shown in corresponding colors.

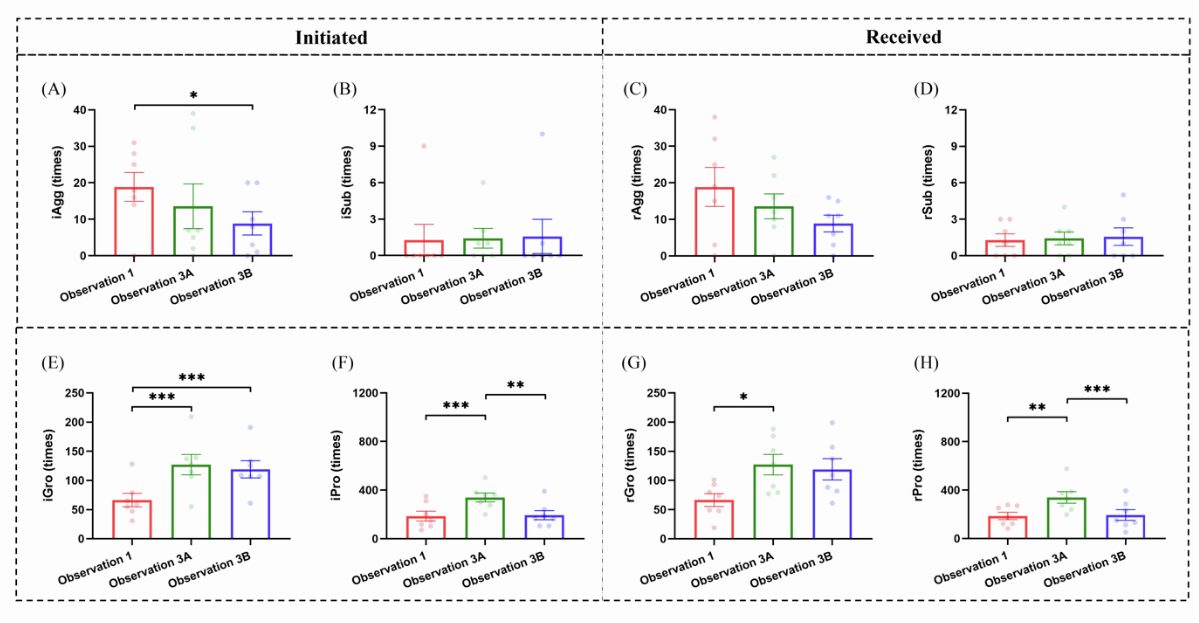

Figure S5. Social behaviors before and after chronic stress in Group BJ (related to Figure 6). (A) and (B) show data of initiated aggressive and submissive behaviors, respectively. (C) and (D) show data of received aggressive and submissive behaviors, respectively. (E) and (F) show data of initiated grooming and proximity, respectively. (G) and (H) show data of received grooming and proximity, respectively. Black * $p < 0.05$, ** $p < 0.01$, *** $p < 0.001$ corrected. Error bars indicate standard error. Individual data points are shown in corresponding colors. Agg: aggressive, Sub: submissive, Gro: grooming, Pro: proximity.

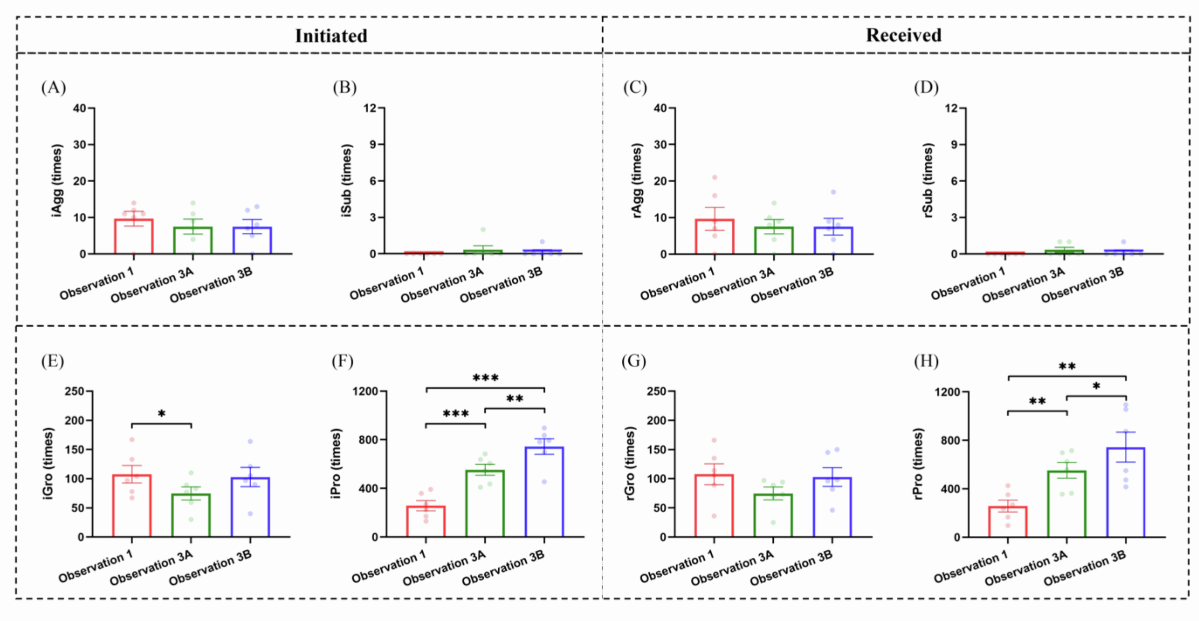

Figure S6. Social behaviors before and after chronic stress in Group SZ (related to Figure 6). (A) and (B) show data of initiated aggressive and submissive behaviors, respectively. (C) and (D) show data of received aggressive and submissive behaviors, respectively. (E) and (F) show data of initiated grooming and proximity, respectively. (G) and (H) show data of received grooming and proximity, respectively. Black * $p < 0.05$, ** $p < 0.01$, *** $p < 0.001$ corrected. Error bars indicate standard error. Individual data points are shown in corresponding colors. Agg: aggressive, Sub: submissive, Gro: grooming, Pro: proximity.
